# Partial Nicotine Reduction and E-Cigarette Users’ Puffing Behaviors Among Adults Aged 21 to 35 Years: A Randomized Crossover Clinical Trial

**DOI:** 10.1001/jamanetworkopen.2024.22954

**Published:** 2024-07-26

**Authors:** Tarana Ferdous, Simanta Roy, Sreshtha Chowdhury, Rime Jebai, Leonardo Maya, Anthony P. DeCaprio, Zoran Bursac, Wasim Maziak

**Affiliations:** 1 Department of Epidemiology, Robert Stempel College of Public Health and Social Work, Florida International University, Miami; 2 Department of Health Law, Policy and Management, School of Public Health, Boston University, Boston, Massachusetts; 3 Forensic and Analytical Toxicology Facility, Global Forensic and Justice Center, Florida International University, Miami; 4 Department of Biostatistics, Robert Stempel College of Public Health and Social Work, Florida International University, Miami

## Abstract

**Importance:**

The advent of salt-based, high-nicotine electronic nicotine delivery systems [e-cigarettes] has contributed to their epidemic use among young people in the US, necessitating the need for policies to address the addictiveness of these products.

**Objective:**

To evaluate the effect of partial nicotine reduction on new-generation e-cigarette users’ puffing behaviors.

**Design, Setting, and Participants:**

This randomized crossover clinical trial was conducted at the Clinical Research Lab for Tobacco Smoking at Florida International University in Miami between April 15, 2022, and October 17, 2023. Using a volunteering sampling method by distributing flyers and advertisements, current e-cigarette users (who preferred 5% nicotine concentration), aged 21 to 35 years, were included.

**Intervention:**

In a crossover design, participants completed 2 sessions of the same product (JUUL or NJOY) that differed by nicotine concentration (3% [JUUL] or 2.4% [NJOY] and 5% [JUUL or NJOY]) in random order. In each session, participants vaped up to 60 minutes ad libitum, preceded by 12 hours of nicotine abstinence.

**Main Outcomes and Measures:**

The primary outcomes were puffing topography parameters (eg, total session time, puffing time, total puffing number, interpuff interval, total inhaled volume, average puff volume, duration, and flow rate) measured during each session and plasma nicotine measured before and after each session.

**Results:**

Among 735 participants who were approached for eligibility, 675 were excluded, and 10 did not complete session 2. Of the 50 remaining current e-cigarette users (mean [SD] age, 23 [3] years; 56% men), 23 (46%) were low nicotine dependent. The median topography parameters were significantly higher during the e-cigarette use sessions with 3% or 2.4% nicotine concentration compared with 5% nicotine concentration for 3 outcomes: puffing time (1.3 minutes [IQR, 0.3-9.4 minutes] vs 1.2 minutes [IQR, 0.2-5.6 minutes]; *P* = .02), puff duration (2.6 seconds [IQR, 0.8-6.9 seconds] vs 2.4 seconds [IQR, 0.4-6.6 seconds]; *P* = .02), and total inhaled volume (1990.0 mL [IQR, 279.0-24 400.0 mL] vs 1490.0 mL [IQR, 148.0-14 300.0 mL]; *P* = .05). The median plasma nicotine boost observed in the 5% nicotine concentration condition (0.0060 mg/L [IQR, 0.0001-0.0249 mg/L]) was significantly higher than that in the 3% or 2.4% session (0.0043 mg/L [IQR, 0.0008-0.0225 mg/L]) (*P* = .001). Additionally, deeper puffing (increased average puff duration and average puff volume) was observed in participants with higher nicotine dependence (1.42 seconds [95% CI, 1.12-1.80 seconds]; *P* = .03) and male users (1.38 mL [95% CI, 1.09-1.75 mL]; *P* = .04) in response to nicotine reduction.

**Conclusions and Relevance:**

This randomized crossover clinical trial provides direct evidence that partial nicotine reduction in salt-based e-cigarettes was associated with acute compensatory puffing and the potential for increased exposure to toxicants. However, given the reduced nicotine delivery associated with nicotine reduction, the acute compensatory response observed in this study may not preclude a population benefit due to the marketing of less addictive products. These results suggest that at least for current e-cigarette users, partial nicotine reduction can lead to enhanced exposure to some toxicants in the short term.

**Trial Registration:**

ClinicalTrials.gov Identifier: NCT05205382

## Key Points

**Question:**

What is the effect of partial nicotine reduction on puffing topography (the characteristics of users’ puffing of electronic nicotine delivery systems [e-cigarettes]) among users aged 21 to 35 years of new-generation nicotine salt–based e-cigarettes?

**Findings:**

This randomized crossover clinical trial that included 50 adult e-cigarette users who preferred using a salt-based, high-nicotine concentration of 5% found that partial nicotine reduction significantly increased users’ topography parameters, including puffing time, puff duration, and inhalation volume.

**Meaning:**

Results of this study provide evidence of compensatory puffing behavior among current e-cigarette users in this population when partial nicotine reduction is applied, suggesting that at least for current e-cigarette users, partial nicotine reduction can lead to enhanced exposure to some toxicants in the short teerm.

## Teaser

This randomized crossover clinical trial assesses the effect of partial nicotine reduction on puffing behaviors among adult users of new-generation nicotine salt–based e-cigarettes.

## Introduction

In recent years, the use of electronic nicotine delivery systems (e-cigarettes) has become an epidemic among young people in the US.^1^ The 2023 National Youth Tobacco Survey showed that 10.0% of high school students and 4.6% of middle school students were current e-cigarette users.^2^ Among these current e-cigarette users, 25.2% were daily users, and 34.7% were frequent users (≥20 days of the past 30 days). Most e-cigarettes contain a high dose of nicotine, which is addictive and causes dependency.^3^ Nicotine also has a toxic effect on pregnant women and their developing fetuses and can negatively affect adolescents’ developing brains.^4,5,6^ Current evidence shows that young people who use high-nicotine e-cigarette products are more likely to accelerate use, become nicotine dependent, and initiate cigarette smoking.^7,8,9,10^

Market analysis and research studies found that the e-cigarette epidemic has been associated with the rise in popularity of new-generation e-cigarette products that efficiently deliver high doses of nicotine. For instance, from 2013 to 2015, e-cigarette product sales with the highest nicotine concentration (4.0%-4.9%) increased from 12.3% to 33.5% but remained under 2% for nicotine-free e-cigarette products.^11^ Additionally, the average nicotine concentration in e-cigarette products increased (from 2.10% to 4.34%) from 2013 to 2018.^12^ Importantly, newer salt-based nicotine formulations, which can deliver nicotine more efficiently to users, have quickly replaced free-base–nicotine e-cigarettes.^13,14,15^ The salt-based e-cigarette products (eg, 5% nicotine JUUL) contributed to 75% of the market share of total e-cigarette products and were considered a significant contributor to a 78% increase in the prevalence of e-cigarette use among US youths from 2017 to 2018.^16,17^ Different studies suggested that salt-based, high-dose nicotine e-cigarettes have contributed to higher nicotine dependence among users and have made it difficult for young adults to quit.^7,16,18^ Interestingly, many e-cigarette users are unaware of the high dose of nicotine exposure from this product.^19,20^ Thus, addressing the addictiveness of e-cigarettes is potentially a major regulatory target to reduce e-cigarette use among young people.

One of the thoroughly researched regulatory targets to reduce the addictiveness and use of tobacco products is nicotine reduction. The US Food and Drug Administration has currently proposed a rule to establish the maximum nicotine levels in many finished tobacco products to reduce their addictiveness.^21^ For cigarette smoking, nicotine reduction has been repeatedly shown to contribute to a reduced daily nicotine intake, smoking of fewer cigarettes, lower levels of dependence, and higher trends in quitting.^22,23,24,25,26,27,28,29^ The compensatory puffing behavior associated with nicotine reduction in cigarettes, moreover, was not substantial and dose dependent, with the lower range of nicotine content showing less compensation and exposure to toxicants.^22,25,29^ Because of the difference in use patterns and delivery of nicotine between e-cigarettes and cigarettes (eg, prolonged intermittent use vs more defined bouts of cigarette smoking, heating e-liquid vs burning tobacco), compensatory puffing behavior associated with nicotine reduction represents an important research question in nicotine reduction strategies for nicotine salt–based e-cigarettes.

The extent and consequences of compensatory puffing in nicotine salt–based e-cigarettes are still largely uninvestigated. What is especially lacking is the use of a sensitive design (eg, within-participant) and standardized assessments that can provide regulation-grade evidence about the potential effects of nicotine reduction on e-cigarette users. This study uses a within-participant crossover design to evaluate the effect of partial nicotine reduction on puffing behavior and plasma nicotine boost in users of nicotine salt–based e-cigarettes and also its relation to users’ nicotine dependence.

## Methods

### Study Overview

This clinical trial used a randomized crossover design and received approval from the Institutional Review Board of Florida International University. Written informed consent was obtained from each participant before the session, and participants were compensated $75 for their time. This study followed the Consolidated Standards of Reporting Trials (CONSORT) reporting guideline for randomized crossover clinical trials. The trial protocol and statistical analysis plan are provided in Supplement 1.^30,31,32^

### Study Participants and Procedures

The study was conducted between April 15, 2022, and October 17, 2023, and included current users of high-nicotine (5%) e-cigarettes (of any brand). Participants were recruited from Miami by distributing flyers, social media advertisements, and word of mouth. Individuals with self-reported chronic health problems, psychiatric conditions, regular use of prescription medications (excluding vitamins or birth control pills), current use of tetrahydrocannabinol, and current use of more than 5 cigarettes or other tobacco or nicotine products per month in the previous year were excluded. Additionally, women who were breastfeeding or tested positive for pregnancy (confirmed through urinalysis) during the screening process were excluded. Participants were randomly assigned into 2 laboratory sessions based on the e-cigarette pods’ nicotine concentration (3% or 2.4% vs 5%) to avoid any order or period effect (eMethods in Supplement 2). After 12 hours of nicotine abstinence, confirmed by expired air carbon monoxide (≤5 ppm) for combustible tobacco use^33^ and baseline plasma nicotine concentration (≤0.005 mg/L) (to convert to micromoles per liter, multiply by 6.164) for noncombustible tobacco use (assessed retrospectively),^34,35,36^ participants underwent a baseline assessment. Each participant then underwent 2 e-cigarette sessions separated by at least 48 hours as a washout period to avoid any carryover effect. Each participant used the same e-cigarette device and brand (JUUL or NJOY) with their preferred flavors in a randomized order: (1) 3% (35 mg/mL) JUUL pod or 2.4% (28 mg/mL) NJOY pod and (2) 5% (59 mg/mL) JUUL pod or 5% (58 mg/mL) NJOY pod. Participants were blinded to the sessions’ conditions (3% or 2.4% and 5%) and were asked to use e-cigarettes ad libitum for up to 60 minutes. Participants were seated in a private room with a comfortable reclining chair, given a choice to use any electronic device, and instructed to use e-cigarettes at their natural pace. Puffing topography data were collected during the sessions, and venous blood samples were collected before and after the sessions.

### Measures

Demographic information (age, sex, race and ethnicity, educational status, employment status, body mass index [BMI], and e-cigarette use-related information [time of first e-cigarette use, device ownership, type of e-cigarette, vaping frequency, pod use, and nicotine-dependence level]) were collected before the session. Race and ethnicity categories, self-reported by participants, included Arab or Middle Eastern, Asian or Pacific Islander, Black, White, Hispanic or Latino, multiracial, and other (including Jewish and Israeli). For the analysis, race and ethnicity were presented as 2 categories: Hispanic or Latino and non-Hispanic or non-Latino (by combining all other race and ethnicity categories except Hispanic or Latino). Race and ethnicity were included in the study because trends of e-cigarette use are known to differ among different racial and ethnic groups.

#### Topography

To measure the puffing behavior of participants, a mouthpiece-connected computerized e-cigarette topography-recording device, eTop (American University of Beirut),^37^ was used during the e-cigarette puffing sessions. The device is a validated instrument with software that can convert pressure signals to airflow (in milliliters per second) data. Data on total session time (minutes), puffing time (minutes), average puff duration (seconds), average flow rate (in milliliters per second), average interpuff interval (seconds), number of puffs, total inhaled volume (in milliliters), average puff volume (in milliliters), and maximum puff volume (in milliliters) were collected. The device was calibrated before each session.

#### Plasma Nicotine

Blood samples were collected before and after the sessions, and after centrifuging, plasma was stored at −80 °C before analysis for nicotine concentration by using liquid chromatography–mass spectrometry (eMethods in Supplement 2).^38^ The plasma nicotine boost for each session was calculated by subtracting the preplasma nicotine value from the postplasma value.

#### Nicotine Dependence

The Penn State Electronic Cigarette Dependence Index was used to measure nicotine dependence for study participants during baseline.^39,40^ It consists of 10 questions on current dependence on e-cigarettes, including users’ urge, quitting difficulty, craving, and other information, rated on a multiple-score scale ranging from 0 to 20 (total scoring: 0 to 3, not dependent; 4 to 8, low dependence; 9 to 12, medium dependence; and ≥13, high dependence). For this analysis, participants were categorized into 2 groups: low dependence (score scale of 0 to 8) and moderate to high dependence (score scale of 9 to 20).

### Sample Size Calculation

All post hoc power calculations were performed with Power Analysis and Sample Size (PASS 2022).^30^ Using a repeated measures analysis of variance *F* test with 1 within (3% or 2.4% vs 5%) and 1 between (moderate to high vs low dependence) factor, a sample size of 50 had at least 80% power at an α level of .05 to detect a medium to large size effect for puffing topography measures (Cohen *f* = 0.4), depending on nicotine concentration conditions (eMethods in Supplement 2).^30,31,32^

### Statistical Analysis

All statistical analyses were conducted using R, version 4.2.1 (R Project for Statistical Computing), using R packages tidyverse, version 1.3.0, for data visualization and gtsummary, version 1.7.2; rstatix, version 0.7.2; and lme4, version 1.1-35.3, for summary and analysis. Statistical significance was assessed using 2-sided tests at the α levels of .10.^41^ The normality of data was assessed using QQ plots (eFigure 1 in Supplement 2). The mean (SD) of continuous variables and the frequencies and proportions of categorical variables were reported. A Wilcoxon signed-rank test was conducted to compare the median (IQR) distributional equality between nicotine pods’ concentration conditions (3% or 2.4% vs 5%) for each outcome (topography parameters and plasma nicotine) separately. We tested only 1 hypothesis by checking the consistency of across-the-board evidence of compensatory puffing rather than highlighting individual differences, as justified by Feise.^42^ Also, the within-participant crossover design controlled individual differences in participants’ overall performance levels, as each participant acted as their control.^43^ A Wilcoxon rank sum test was conducted to compare the distributional equality of all outcomes between 2 study orders (starting with 3% or 2.4% and 5%) to check the carryover effects (eTable 1 in Supplement 2).

As a secondary analysis, linear mixed models were conducted for nicotine reduction sessions to predict the change in all puffing topography and plasma nicotine outcomes at different levels of users’ nicotine dependency and sex. Furthermore, in the analysis, we adjusted for confounders, such as age, sex (excluded for the secondary analysis for sex), race and ethnicity, nicotine dependence (excluded for the secondary analysis for users’ nicotine dependency), BMI, session time, and device type. Model assumptions were checked for residual normality, heteroscedasticity, and multicollinearity (eMethods, eTable 2, and eFigure 2 in Supplement 2).

## Results

In total, 735 participants were approached for telephone screening, and 675 were excluded (513 did not meet inclusion criteria, 43 declined, and 119 had other reasons) (Figure). Among the eligible 60 participants, 10 did not complete session 2 (4 for order 1, which started with the 3% or 2.4% condition, and 6 for order 2, which started with the 5% condition), and data analysis included 50 participants who completed both sessions (mean [SD] age, 23 [3] years; 22 women [44%]; 28 men [56%]).

**Figure.**
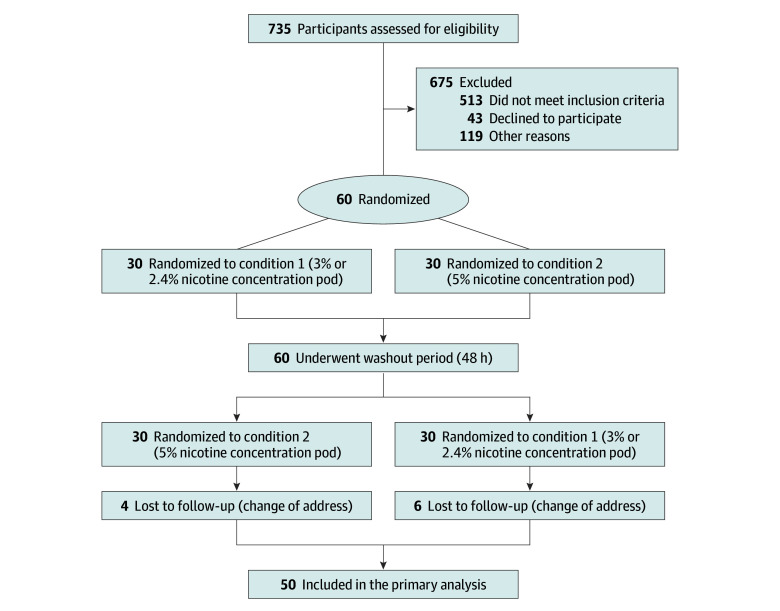
CONSORT Diagram CONSORT indicates Consolidated Standards of Reporting Trials.

For the 50 participants, the mean (SD) BMI (calculated as weight in kilograms divided by height in meters squared) was 27 (7.0) and was equally distributed between men and women and e-cigarette dependence (low and moderate to high) groups. More than half of the participants were Hispanic or Latino (68% [n = 34]) and currently employed (58% [n = 29]); 16 (32%) were non-Hispanic or non-Latino. Most of the study participants owned an e-cigarette device (90% [n = 45]), vaped daily (72% [n = 36]), and had been using it for more than 1 year (90% [n = 45]); 23 (46%) had low nicotine dependence (Table 1).

**Table 1.** Characteristics of the Study Participants by Sociodemographics and E-Cigarette Use

Characteristic	Participants, No. (%) (N = 50)
Age, mean (SD), y	23 (3)
Sex	
Female	22 (44)
Male	28 (56)
Race and ethnicity	
Hispanic or Latino	34 (68)
Non-Hispanic or non-Latino^a^	16 (32)
Educational status	
Bachelor’s degree or more	25 (50)
Some college education (no degree) or less	25 (50)
BMI, mean (SD)	27 (7.0)
Current employment status	
Employed (full-time or part-time)	29 (58)
Not employed	21 (42)
First e-cigarette use	
<1 y Ago	5 (10)
≥1 y Ago	45 (90)
Own e-cigarette device	
Yes	45 (90)
No	5 (10)
Type of current e-cigarette use of any brand	
Disposable	46 (92)
Nondisposable, refillable, tank, or other	4 (8)
Vaping d in past 6 mo	
Every day	36 (72)
1 Per wk	14 (28)
Vaping times per d^b^	
<20	32 (64)
≥20	18 (36)
No. of pods used per wk, mean (SD)	1.3 (1.0)
Nicotine dependence level	
Low	23 (46)
Moderate to high	27 (54)

Abbreviation: BMI, body mass index (calculated as weight in kilograms divided by height in meters squared).

^a^ Included the following combined self-reported races and ethnicities: Arab or Middle Eastern, Asian or Pacific Islander, Black, White, multiracial, and other (including Jewish and Israeli).

^b^ One time = 15 puffs.

### Puffing Topography and Plasma Nicotine Measures by Nicotine Concentration Conditions

During a 60-minute ad libitum e-cigarette–use session with nicotine concentration conditions (3% or 2.4% vs 5%), a higher value was observed for an e-cigarette–use session with 3% or 2.4% nicotine concentration compared with 5% for most topography parameters. For example, median puffing time (1.3 minutes [IQR, 0.3-9.4 minutes] vs 1.2 minutes [IQR, 0.2-5.6 minutes]; *P* = .02), puff duration (2.6 seconds [IQR, 0.8-6.9 seconds] vs 2.4 seconds [IQR, 0.4-6.6 seconds]; *P* = .02), and total inhaled volume (1990.0 mL [IQR, 279.0-24 400.0 mL] vs 1490.0 mL [IQR, 148.0-14 300.0 mL]; *P* = .05) were significantly higher when participants used a 3% or 2.4% nicotine content e-cigarette compared with an e-cigarette with 5% content (Table 2). The median plasma nicotine boost observed in the 5% nicotine concentration condition (0.0060 mg/L [IQR, 0.0001-0.0249 mg/L]) was significantly higher than that in the 3% or 2.4% session (0.0043 mg/L [IQR, 0.0008-0.0225 mg/L]) (*P* = .001) (Table 3).

**Table 2.** E-Cigarette Users’ Topography by Nicotine Concentration^a^

Parameter	Sessions with different nicotine concentration conditions, median (IQR) (N = 50)		*P* value^b^
	3% or 2.4% Nicotine	5% Nicotine	
Total session time, min	62.2 (31.3-75.5)	61.8 (31.2-68.7)	.68
Puffing time, min	1.3 (0.3-9.4)	1.2 (0.2-5.6)	.02
Average puff duration, s	2.6 (0.8-6.9)	2.4 (0.4-6.6)	.02
Average flow rate, mL/s	22.0 (9.9-58.0)	22.9 (12.5-62.8)	.69
Average interpuff interval, s	91.9 (4.7-748.0)	107.0 (15.3-527.0)	.85
No. of puffs	32.0 (5.0-594.0)	27.5 (6.0-238.0)	.35
Total inhaled volume, mL	1990.0 (279.0-24 400.0)	1490.0 (148.0-14 300.0)	.05
Average puff volume, mL	60.3 (17.9-271.0)	60.2 (10.1-200.0)	.14
Maximum puff volume, mL	84.5 (27.7-454.0)	87.4 (22.3-328.0)	.09

^a^ Normality was assessed using the QQ plot for all outcomes by each session group, and distributions were not normal.

^b^ The Wilcoxon signed rank test was used.

**Table 3.** E-Cigarette Users’ Plasma Nicotine by Nicotine Concentration^a^

Plasma nicotine measures, mg/L	Sessions with different nicotine concentration conditions, median (IQR) (N = 30)^b^		*P* value^c^
	3% or 2.4% Nicotine	5% Nicotine	
Before the session	0 (0-0.0041)	0 (0-0.0041)	.89
After the session	0.0048 (0.0008-0.0253)	0.0066 (0.0010-0.0267)	.002
Plasma nicotine boost (after to before the session)	0.0043 (0.0008-0.0225)	0.0060 (0.0001-0.0249)	.001

SI conversion factor: To convert milligrams per liter to micromoles per liter, multiply by 6.164.

^a^ Normality was assessed using the QQ plot for all outcomes by each session group, and distributions were not normal.

^b^ Not analyzed for 20 participants (under laboratory analysis, had >0.0050 mg/L plasma nicotine before sessions, or unable to collect blood after sessions).

^c^ The Wilcoxon signed rank test was used.

### Puff Topography and Plasma Nicotine Boost by Dependence, Sex, and Nicotine Concentration Conditions

Results from adjusted models showed that in sessions with 3% or 2.4% nicotine (vs 5% nicotine), compared with e-cigarette users with low dependence, those with moderate to high dependence had deeper puffing behavior, as they exhibited 42% higher average puff duration (1.42 seconds [95% CI, 1.12-1.80 seconds]; *P* = .03) and 19% higher average puff volume (1.19 mL [95% CI, 0.92-1.52 mL]; *P* = .31). Additionally, male users had a deeper puffing behavior, with a 38% higher average puff volume (1.38 mL [95% CI, 1.09-1.75 mL]; *P* = .04) than women (Table 4). No multicollinearity or heteroscedasticity was detected (eTable 2 in Supplement 2).

**Table 4.** Comparisons of Nicotine Dependence and Sex Among E-Cigarette Users

Outcome	β estimate (95% CI)								
	Puffing time, min	Average puff duration, s	Average flow rate, mL/s	Average interpuff interval, s	No. of puffs	Total inhaled volume, mL	Average puff volume, mL	Maximum puff volume, mL	Plasma nicotine boost, mg/L^a^
Dependence^b^									
Low	1 [Reference]	1 [Reference]	1 [Reference]	1 [Reference]	1 [Reference]	1 [Reference]	1 [Reference]	1 [Reference]	1 [Reference]
Moderate to high	0.04 (−0.33 to 0.40)	0.35 (0.11 to 0.59)	−0.18 (−0.31 to −0.04)	0.35 (−4.32 to 0.75)	−0.31 (−0.70 to 0.08)	−0.14 (−0.56 to 0.28)	0.17 (−0.08 to 0.42)	0.09 (−0.18 to 0.36)	0.0053 (0.0001 to 0.0010)
Exponentiatial^c^	1.04 (0.72 to 1.52)	1.42 (1.12 to 1.80)	0.83 (0.73 to 0.96)	1.42 (0.01 to 2.12)	0.73 (0.50 to 1.08)	0.87 (0.57 to 1.32)	1.19 (0.92 to 1.52)	1.09 (0.83 to 1.43)	0.0017 (0.0011 to 0.0027)
*P* value	.86	.03	.05	.18	.23	.61	.31	.60	.11
Sex^d^									
Female	1 [Reference]	1 [Reference]	1 [Reference]	1 [Reference]	1 [Reference]	1 [Reference]	1 [Reference]	1 [Reference]	1 [Reference]
Male	−0.15 (−0.49 to 0.20)	0.20 (−0.02 to 0.42)	0.12 (−0.003 to 0.24)	0.36 (−1.15 to 0.73)	−0.35 (−0.71 to 0.01)	−0.03 (−0.41 to 0.36)	0.32 (0.09 to 0.56)	0.19 (−0.05 to 0.44)	−0.0004 (−0.0008 to 0.0001)
Exponentiatial^c^	0.86 (0.61 to 1.22)	1.22 (0.98 to 1.52)	1.13 (1.00 to 1.27)	1.43 (0.32 to 2.07)	0.70 (0.49 to 1.01)	0.97 (0.66 to 1.43)	1.38 (1.09 to 1.75)	1.38 (1.09 to 1.75)	0.0007 (0.0004 to 0.0011)
*P* value	.51	.16	.14	.13	.14	.91	.04	.23	.21

SI conversion factor: To convert milligrams per liter to micromoles per liter, multiply by 6.164.

^a^ Not analyzed for 20 participants (under laboratory analysis, had >0.0050 mg/L plasma nicotine before sessions, or unable to collect blood after sessions).

^b^ A linear mixed model analysis was used after adjusting for participants’ age, sex, race and ethnicity, body mass index, device type, and total session time of study participants.

^c^ Exponentiated β coefficients (log-transformed β estimate).

^d^ Linear mixed model analysis after adjusting for participants’ age, race and ethnicity, body mass index, device type, nicotine dependence, and total session time of study participants.

## Discussion

Given the epidemic use of new nicotine salt–based e-cigarettes among young people in the US and the need to regulate their high nicotine content and addictiveness, this randomized crossover clinical trial is the first, to our knowledge, to evaluate the effect of partial nicotine reduction on users’ puffing behaviors using a within-participant crossover design. The higher puffing topography parameters observed with the lower nicotine concentration (3% or 2.4%) session compared with participants’ regular nicotine (5%) session provided direct evidence of e-cigarette users’ compensatory puffing in response to partial nicotine reduction. However, these enhanced puffing patterns were not enough to achieve similar plasma nicotine boost levels, in which the regular nicotine potency session delivered significantly higher levels of nicotine. Additionally, e-cigarette users with moderate to high nicotine dependence had significantly deeper puffing behavior and plasma nicotine boost in response to lowering nicotine compared with low-dependence users. Although based only on an acute effect model, these findings raise the possibility that partial nicotine reduction can lead to more toxicant exposure, at least short term, due to enhanced puffing patterns. This acute compensatory response, however, does not preclude a population benefit (ie, nicotine-naive users) due to the marketing of less addictive products.

Previous studies on puffing behavior due to nicotine reduction among e-cigarette users have shown mixed results. For example, several studies have shown increased topography parameters among e-cigarette users due to nicotine reduction.^44,45,46,47,48,49,50^ However, all of these studies have used older generations of e-cigarettes (with free-base nicotine at 18 to 36 mg/mL as the highest nicotine concentration) rather than newer nicotine salt–based e-cigarettes (with 58 to 59 mg/mL as the nicotine concentration), which deliver more and faster nicotine to users. In addition, these studies suffered from considerable limitations that precluded making informed judgments about the effect of nicotine reduction on e-cigarette users.^44,45,46,47,48,49^ For example, limiting puffing protocol to 10 puffs^48^ is inconsistent with natural patterns of e-cigarette use characterized by intermittent puffing over extended periods (unlike the distinct bouts of cigarette smoking). In addition, some studies^47,48^ required short nicotine-abstinence periods (1 hour), which is inadequate to exclude the effect of prior nicotine use on study parameters given nicotine bioavailability and metabolism (2-hour half-life).^36^ Other studies did not include e-cigarette users^44^ or the same e-cigarette product for different sessions of the same participant,^47^ which makes it difficult to interpret their results in terms of inferring the noticed changes in puffing patterns in response to nicotine reduction. We tried to overcome these shortcomings by using a well-validated puff topography device,^37^ allowing ad libitum puffing for up to 60 minutes, using the same brand of e-cigarette for both sessions in each participant, recruiting only current e-cigarette users, and requiring 12-hour plasma nicotine-validated abstinence before each study session.

The US Food and Drug Administration is currently considering nicotine reduction as an important regulatory route to reduce the addictiveness of tobacco and nicotine products. To do so, evidence-based guidance about the effect of different levels of nicotine reduction on users’ behavior and exposure to toxicants is needed. Such knowledge is available for cigarette smoking. For example, Berman and Glasser^51^ examined, in a systematic review topography, patterns and toxicant exposures associated with the smoking of low nicotine content cigarettes compared with conventional cigarettes. The review showed that smoking cigarettes with moderately low nicotine content (8.4 to 11.7 mg nicotine per gram of tobacco) was associated with higher exposure to toxicants compared with conventional cigarettes (15.5 to 18.0 mg nicotine per gram of tobacco) due to compensatory puffing. However, smoking very low nicotine cigarettes (≤0.4 mg nicotine per gram of tobacco) was associated with lower topographical parameters and exposure to toxicants compared with conventional cigarettes (15.5 to 18.0 mg per gram of tobacco). Given the obvious differences in access, use patterns, and composition of the inhaled mixture between e-cigarettes and conventional cigarettes, we could not extrapolate the expected effect of reducing nicotine for e-cigarettes based on the cigarette literature. Our results showed comparable compensatory puffing patterns in response to partial nicotine reduction that reflected moderate nicotine reduction in cigarette smokers. The next step is to assess whether this enhanced puffing is associated with increased exposure to toxicants and the effects of further reductions in nicotine (very low) on e-cigarette users’ puffing behavior and exposure to toxicants. One of the reasons why we expect to see deviance in e-cigarette responses to low nicotine levels is that e-cigarette aerosol is less irritant compared with cigarette smoke. This would likely affect the ability of users of these different products to sustain intensive puffing patterns in response to very low nicotine levels.

Despite our documented enhanced topography patterns associated with partial nicotine reduction compared with regular potency, these fell short of achieving a comparable plasma nicotine boost. This finding is corroborated by studies in cigarette smokers as well as studies of early generations of e-cigarettes, in which the plasma nicotine or cotinine of participants progressively declined as the nicotine contents of these tobacco products were lowered.^23,24,25,26,44,45,46,47,48,49,50^ Thus, although nicotine yield is likely the main driver for enhanced puffing associated with lowing nicotine, it did not lead to achieving the same level for participants’ usual concentration. Looking at the differences in puffing patterns in response to partial nicotine reduction according to dependence levels provides some insight into current e-cigarette users’ main puffing adaptations in response to nicotine content. Our study showed that taking deeper and more prolonged puffs was the most prominent adaptation among the users with moderate to high dependence in response to nicotine reduction compared with less-dependent users. This is important, as it will likely affect the yield of the toxic effect in a unique way that is specific to this tobacco-use method, since puffing parameters have differential effects on aerosolized toxicants.^52,53^ Similar differences were noticed according to sex, although they did not reach statistical significance. Additionally, in response to nicotine reduction, men in our study took larger and longer puffs than did women. These dynamics need to be investigated further, especially in terms of their effect on exposure to different toxicants.

### Strengths and Limitations

Our study has strengths of using a within-participant crossover design, a nicotine salt–based e-cigarette (fourth generation), current e-cigarette users with 5% nicotine preference, strictly enforced 12-hour abstinence (through plasma nicotine measures) prior to each session, and counterbalancing the order of sessions with at least 48 hours of a washout period between them. The enforcement of 12-hour abstinence and exclusion of e-cigarette users who used them with other addictive products (eg, tetrahydrocannabinol) ensured that the detected differences were mainly due to the nicotine concentration condition. These methods helped substantiate the robustness of our findings and remove the potential effects of session order, carryover, device, and nicotine preference on the study results. Although this research needs to be supplemented by studying the effects of further reductions in nicotine (very low) on puffing responses and associated exposures, we believe that assessing partial nicotine reduction is important given that several countries, including the US, are considering partial nicotine reduction to limit the addictiveness of e-cigarettes (eg, similar to the European Union that limits nicotine concentration to 20 mg/mL or approximately 2% in e-cigarettes).^54^

This study’s limitations include the laboratory setting environment and the use of limited e-cigarette products, which can affect users’ vaping behavior. However, our study’s laboratory setting was necessary to make standardized assessments that enabled valid comparisons between sessions’ conditions and to ensure that the detected differences were mainly due to the study conditions. Such laboratory methods have been the mainstay in studying emerging tobacco products for regulatory purposes.^45,46,47,48,49,50,51^ The selection of the 2 e-cigarette brands was mainly due to the availability of nicotine concentrations needed for this study and their likelihood of being more standardized compared with other e-cigarette brands available in the market. We also kept the same device constant through both sessions for each participant. This helped to ensure that all study parameters were constant between the 2 sessions except for the nicotine concentration. Next, by using an acute laboratory model, our study did not answer the question about the long-term effects of partial nicotine reduction on e-cigarette users’ puffing patterns and possible exposure to toxicants or whether the detected changes were sustainable with repeated use. Additionally, our model was not designed to address the issue of statistically significant vs clinically relevant differences in topography outcomes. However, we can argue that our results were of potential clinical value in light of the existing literature about nicotine reduction in cigarettes^52^ and earlier-generation e-cigarettes.^46^ These studies show a correlation between puff topography changes in response to nicotine reduction similar to ours and toxicant exposure.^45,49,50^ Lastly, our study results applied mostly to the users’ characteristics described in the study. However, we do not believe that this precludes their broader implications for other e-cigarette users with nicotine addiction, given the large amount of literature about nicotine dependence, self-medication, and nicotine levels.^55,56^

## Conclusions

This randomized crossover clinical trial showed that partially lowering nicotine concentrations led to acute compensatory puffing in nicotine salt–based e-cigarettes among users aged 21 to 35 years. Such a response can predict higher exposure to at least some toxicants in the short term among current e-cigarette users who are moderately to highly dependent. However, given the reduced nicotine delivery associated with nicotine reduction, the acute compensatory response observed may not preclude a population benefit due to the marketing of less addictive e-cigarettes. Further research can aid in understanding the persistence of such an effect and how this compensatory puffing influences exposure to various toxicants. As important is studying the impact of further reduction in nicotine on users’ puffing behaviors and toxicant exposure.

## Notes

Supplement 1.
Trial Protocol

Supplement 2.
eMethods
**eFigure 1.** QQ Plot to Assess the Normality of All Outcomes in Table 2 and Table 3 by Each Session Group (3% vs 5%): Shown Only for Topographic Outcomes **eFigure 2.** QQ Plot to Assess the Normality of Residuals for All Outcome Variables (Untransformed vs. Log-Transformed): Shown Only for Models With “Nicotine Dependence Levels” as the Independent Variable **eTable 1.** E-Cigarette Users’ Topography Measured by Nicotine Concentration Session Orders (Order 1 Started With 3%, and Order 2 Started With 5%) (n = 100): Shown Only for Topographic Outcomes **eTable 2.** Test for Normality and Heteroscedasticity for Model Residuals and Multicollinearity for Variables (Untransformed and Log-Transformed Outcome Variables): Shown Only for Adjusted Models With “Nicotine Dependence Levels” As the Independent Variable
eReferences

Supplement 3.
Data Sharing Statement
