# Supplementary material for: Partial Nicotine Reduction and E-Cigarette Users’ Puffing Behaviors Among Adults Aged 21 to 35 Years: A Randomized Crossover Clinical Trial
Source: JAMA Netw Open. 2024 Jul 26;7(7):e2422954. doi: 10.1001/jamanetworkopen.2024.22954 (PMC11282440; doi:10.1001/jamanetworkopen.2024.22954)
Supplement: Supplement 1. — Trial Protocol [file jamanetwopen-e2422954-s001.pdf]

Study Protocol

Assessing the effect of nicotine reduction on ENDS users' addiction and exposure

Background

The use of electronic nicotine delivery systems (ENDS) has reached epidemic levels among young people in the US. While estimates vary, ENDS have become the leading tobacco product used by young people in this country. ENDS heat and vaporize a nicotine-containing liquid to produce an inhalable aerosol mist. In addition to dependence-producing nicotine, ENDS emit other toxicants including aldehydes/carbonyls, a class of potent respiratory toxicants implicated in most non-cancer pulmonary diseases in cigarette smokers. Evidence indicates that young people using ENDS are likely to accelerate use, become nicotine dependent (ND), and initiate cigarette smoking. Epidemiological studies and market analysis, moreover, suggest that "pod-mod" ENDS, JUUL in particular, had the biggest impact on the ENDS epidemic among young people. Unlike older ENDS generations, JUUL pioneered the use of nicotine salts, which allowed the delivery of high doses of nicotine in a smooth (protonated) form to users. Thus, addressing the addictiveness of ENDS through nicotine reduction (NR) can be a major regulatory strategy to reduce ENDS use among young people. In cigarette smokers, several observational studies and clinical trials have shown that smokers who switch to low-nicotine cigarettes reduce daily nicotine intake, smoke fewer cigarettes, report lower dependence, and show higher trends in quitting. Similar patterns are expected in ENDS users, as our preliminary study of NR in young JUUL users shows that it leads to decreased satisfaction, dependence suppression, and intention to use in the future. We also observed significant differences in puff topography parameters [i.e. mean (SD) of total inhaled volume 2.9 (1.5) vs. 1.6 (0.7) L: $p = 0.03$] consistent with compensatory puffing behavior in response to lower nicotine concentration (3% vs 5%). However, reducing ENDS nicotine levels carries the risk of compensatory puffing and increased exposure. Evidence suggests that such compensation is dose and ND related, where very low nicotine products and beginning users are likely to have minimal compensation. Accordingly, assessing the potential role of NR regulations to reduce ENDS use and addiction requires standardized comparisons involving a range of NR levels and ENDS users at different stages of their use trajectory. It also requires assessing a wide array of outcomes to evaluate the effects of compensatory puffing on exposure to key pulmonary toxicants. These goals can be achieved by applying within-between subject comparisons to assess responses to a range of NR levels (partial vs. total) in ENDS users with different use profiles. In this study we plan to compare among ENDS users, the effect of full nicotine concentration (5%), partial (3%) or nicotine-free, 0% nicotine reduction (NR) on users' dependence, satisfaction, clinical (e.g. BP, oximetry, lung functions, symptoms), puffing behavior, and exposure to toxicants. We will recruit current ENDS users (n=120; 21-35 yrs), for a 3X2 within subject crossover lab study. Because the effect of NR on ENDS users will likely vary by dependence and stage of ENDS use, we will study NR effects in high vs. low-dependence users. Therefore, the 3 nicotine conditions (5%, 3%, 0%) x 2 times (pre-post) are the within-subject factors, and they will be stratified by the nicotine dependence in the analysis.

Study Aims

Aim 1: To assess puffing behavior in response to NR on ENDS users. Under the same protocol, puff topography (e.g. puff volume, puff frequency, duration) will be assessed continuously during the 3 sessions, while plasma nicotine will be measured pre-post sessions to assess nicotine boost and its correlation to puffing parameters. This aim will help understand users' compensatory puffing in response to partial and full NR and its effect on plasma nicotine.

Aim 2. To assess exposure to toxicants associated with NR among ENDS users. We will measure toxicant emissions (14 aldehydes) in the analytical lab using a smoking robot that reproduces the puff- by-puff behavior (playback) of each participant for all three nicotine conditions. This aim will reveal how compensatory puffing behavior in response to NR influences acute exposure to pulmonary toxicants.

Aim 3: To assess the subjective and clinical effects of NR on ENDS users. This study will focus on pre-post-use assessment of craving, withdrawal, satisfaction, intention to quit or use in the future, and clinical symptoms (e.g. dry mouth, eye irritation, palpitation, nausea). This aim will show ENDS users' response to NR on product satisfaction, dependence, and clinical symptoms.

The proposed studies will give clear evidence on the potential of NR regulations to limit the addictiveness and use of ENDS, and to help predict any potential side effects of NR on ENDS users.

Study Design

This study is a 3X2X2 within-between subject crossover lab study. Within-subject factors are the 3 nicotine concentration conditions (0%, 3%, 5%) x 2 time (pre-post use), while X 2 high vs. low frequency use is the between-subject factor. Participants will undergo 3 ENDS use sessions that differ by nicotine concentration (0%, 3%, 5%) and are preceded by 12-hour abstinence, with session order counter-balanced and separated by at least 48 hours to mitigate order and carryover effects. Pre- and post-use venous blood sampling will assess blood nicotine boost according to the 3 conditions. Also, pre-post- assessment of craving, withdrawal, satisfaction, intention to quit or use in the future, lung functions, and clinical symptoms will be conducted (Aim 1). Puff topography parameters (e.g. puff number, volume, and duration, inter puff interval) will be assessed during ad libitum use sessions of up to 1 hour each (as in Vargas-Rivera et al., 2020) (Aim 2). Playback of these puff topography sessions will be used to model exposure to toxicants (aldehydes, nicotine) according to the 3 nicotine conditions (Aim 3). A follow-up phone call at 3-month post-study will be made to evaluate nicotine preferences and changes in ENDS and tobacco use.

Sample size

All power calculations were performed with PASS19 (PASS, 2019). We built our calculation of the sample size for this study based on our preliminary study of the effect of NR on subjective (e.g. craving, urges) and puff topography measures (pls. see Preliminary Studies), where medium effect size (Cohen's f=0.2) depending on nicotine condition was detected. Expecting a maximum 20% loss due to noncompliance with abstinence or study protocol (Vargas-Rivera et al., 2020), this study will include a dropout inflated sample of 150 ENDS users. Using repeated measures ANOVA F-test with 2 within and 1 between factors, a total dropout adjusted sample size of 120 (2X60) participants will have at least 80% power to detect small to medium-size effect (Cohen's f=0.1-0.2) or larger for 2 within-subject (NR condition and time) and 1 between subject (use frequency) factors, as well as their interactions at 0.05 level of significance, assuming sphericity and a moderate correlation (0.5) among repeated measures (Cohen, 1988).

Participants and recruitment

Participants: We will recruit 120 current ENDS users defined as using ENDS either daily or occasionally in the past 30 days (as in Vargas-Rivera et al., 2020). Individuals who report smoking cigarettes or other tobacco/nicotine products will be included to increase the generalizability of results and enhance recruitment provided that they don't use these tobacco products >5 times/past month (as in Ben Taleb et al., 2020). As our study focuses on nicotine dependence related parameters, any substantial use of other tobacco/nicotine products will likely bias our planned assessments. Similarly, the pre-study 12 hours abstinence period from all tobacco/nicotine products and using the same product type/brand ensures that study outcomes reflect mainly each session's condition. We aim to recruit equal numbers of men and women.

Inclusion and exclusion criteria: Participants need to be generally healthy, between 21-35 years old, provide written informed consent, and agree to attend the lab on two occasions and use their ENDS product according to the study protocol. Participants will be asked to abstain from ENDS and all tobacco/nicotine products use for at least 12 hours prior to each session. The abstinence period is needed to clear nicotine from prior-to-study use and ensure that all study measures are influenced mostly by the study conditions. Testing short-term abstinence of ENDS, which does not produce carbon monoxide (CO) is an ongoing challenge (Hiler et al., 2017). Therefore, we will follow published recommendations (Blank et al., 2016) to use the gold standard of plasma nicotine to eliminate data from non-compliant participants later. Because this will entail a loss of data, we will try to minimize that and improve compliance by asking the participants to arrive at the scheduled study sessions one hour early for an observation period, and complete a CO test (Blank et al., 2016). The CO test will be good to detect combustible tobacco products' use (e.g. cigarette smoking), and act as a bogus pipeline for ENDS use (Blank et al., 2016). This procedure has been shown to cut participants' non-adherence to the abstinence condition to <10% (Spindle et al., 2018), which is consistent with our own experience (Vargas-Rivera et al., 2020). A blood draw will be completed before each session, abstinence will be confirmed using plasma nicotine levels less than 5 ng/ml (Benowitz, Hukkanen, & Jacob, 2009). Individuals with a self-reported history of chronic disease, psychiatric conditions, history of cardiovascular disease, low or high blood pressure (BP) (systolic BP>150 mm Hg, or diastolic BP>100 mm Hg), seizures, or regular use of prescription medications (other than vitamins or birth control) will be excluded (St Helen et al., 2017). This will be assessed by history and physical examination conducted by the research nurse during a screening visit. The research nurse will have access to an on-call Medical Monitor (physician) in case of concern or for consultation at any point during the study (as is the current practice in our lab). The physical examination will include measuring BP, heart rate (HR), temperature, pulse oximetry, and weight/height. Importantly, considering the current COVID-19 pandemic we will follow CDC and FDA guidelines for clinical research and COVID-19 to minimize any potential risk of infection transmission as detailed in our Human Subject Protection part (CDC, 2020b; FDA, 2020c).
Briefly, we will apply a protocol consisting of 5 main areas;
1-Screening of Participants for COVID-19;
2- Social and Physical Distancing;
3- Use of Personal Protective Equipment (PPE);
4- Cleaning and disinfecting;
5- Personal Hygiene and Hand Washing.

Participants with symptoms/history suggestive of COVID-19 (e.g. □100.4°F, cough, contact) or with a history of cannabis use (risk for e-cigarette or vaping product use-associated lung injury-EVALI) will be excluded from participation (details in Human Subjects Protection). As knowledge about COVID-19 is rapidly evolving we will apply best practices at the time of study to protect human subjects and our staff (e.g. require vaccination record if such becomes feasible). FIU has started providing vaccination to all research staff with direct contact with human subjects, and free-of-charge COVID-19 testing facilities have become available within and in the vicinity of FIU (e.g.FIU-Curative., 2020). Women will be excluded if they are breast-feeding or test positive for pregnancy (by urine pregnancy testing) at screening (St Helen et al., 2017). Finally, we will also exclude individuals if they intend to quit e-cigarette within the next 3 month and if they use THC in e-cigarette.

Recruitment: We will recruit participants using a combination of online, offline, and in-person recruitment methods to ensure successful recruitment (Buller et al., 2012). Offline items such as posters, flyers, and study inserts will be posted and distributed on FIU and other university campuses in Miami (e.g. University of Miami), nearby off-campus locations, and around ENDS shops in Miami. Online recruitment will include student listservs, Facebook, and other social media. In-person recruitment will involve handing out flyers and word-of-mouth targeting vape shops in Miami. Recruitment materials will be Institutional Review Board (IRB) approved and will have contact information (phone, e-mail) for initial eligibility screening. Based on previous experience, we expect to study 10 subjects/month (Vargas-Rivera et al., 2020). We will continue recruitment until we reach the target sample (120 with complete sessions). While recruitment is always a challenge, Maziak's lab has easily recruited JUUL users in the past year with negligible drop out for 2 session protocol using comparable incentives, to the extent that wehad to turn down many eligible participants due to sampling saturation (Vargas- Rivera et al., 2020). This is due to the widespread use of ENDS, and JUUL among young adults, especially in a large university with about 57,000 students such as FIU, where our lab is located. Participants completing the on-site screening and 3 lab sessions, and 3-month phone follow-up will be compensated a total $200 at the end of the study to encourage completeness: $50 after completion of each clinic session ($150) and $50 for the follow up phone call.

Screening, and informed consent: Individuals who are identified as potentially eligible based on the phone screening will be asked to attend an onsite-screening, which will involve assessment of inclusion/exclusion criteria, physical examination, COVID-19 screening, and urine pregnancy testing for women. Prior to screening, potential participants will undergo a review of the study and consent procedures to ensure that they understand the study, its risks/benefits, and their rights as research participants. Consented (written) individuals will then undergo a physical examination conducted by the research nurse, who will have access to an on-call Medical Monitor (physician) in case of concern or for consultation at any point of the study as it is the standard practice in our lab. Successfully consented participants will be scheduled for their 1st session and asked to abstain from all tobacco/nicotine use 12 hours prior to it.

Experimental protocol

Participants from each frequency group will be randomly assigned to one of the nicotine concentration conditions and attend the lab for two, ~2-h sessions, separated by a 48-h washout period, and differ by nicotine concentration (3%,5% 0%). Session order will be counterbalanced to mitigate order and carry-over effects. At the beginning of the 1st session, participants will provide consent, complete demographic and personal information including age, sex, socioeconomic status, race/ethnicity, and detailed tobacco/nicotine use history. Participants will get familiarized with the study procedures, measures, and they will be given time to adapt to the lab. After adaptation, continuous measurement of physiological responses begins, and 10 ml of venous blood is sampled, lung function tests will be conducted, and participants are asked to respond to subjective measures (pls. see below Outcome Measures). Participants will inhale on their session ENDS ad libitum for up to 60 minutes (as in Vargas-Rivera et al., 2020). At the end of the ENDS use period 10 ml of blood will be sampled, lung function tests repeated, and subjective measures assessed. The session will terminate 30 minutes after the last puff, and the 2nd session is scheduled. A follow-up phone call at 3-month post-study will be made to evaluate nicotine preferences and changes in ENDS and tobacco use.

Outcome measures

We will utilize our clinical lab model to study the effect of NR on the following outcomes assessed according to the plan described in Table 1. All assessments will be based on instruments that are standard in clinical lab studies of addictive behaviors modified for the ENDS. Participants will use a tablet to respond to these measures using RedCap. Briefly, we will assess:

1. Demographic and baseline characteristics; including age, race, sex, and reasons for ENDS use. Other characteristics will include the frequency of ENDS use, and the history of other tobacco/nicotine product use
2. Dependence measures include; 1) Use satisfaction (Modified Cigarette Evaluation Questionnaire (mCEQ); 2) Dependence (Questionnaire of Smoking Urges-brief and the Minnesota Nicotine Withdrawal Scale, Penn State Electronic Cigarette Dependence Index; 3) Puff sensory effects (Duke Sensory Scale).
3. Use related measures including 1) Harm perception 2) Intention to quit or use in the future; 3) Reasons for ENDS use, and 4) Visual Analogue Scale (VAS) to measure ENDS use experience such as pleasantness, enjoyment, and pleasure from use.
4. Clinical symptoms, such as (dry mouth, eye irritation, palpitations, and nausea) will be assessed pre-post ENDS use using standard clinical assessments. Symptoms like cough, sore throat will be assessed after-session since our participants will be free of these symptoms pre-session to exclude potential COVID-19 cases (pls. see Inclusion and Exclusion criteria).
5. Cardiovascular measures: To monitor participants' vital signs, physiological measures will be monitored during each session such as heart rate, blood pressure, and pulse oximetry using the Noninvasive Patient Monitor 507E, Criticare Systems, Waukesha, WI.
6. Puff topography: Puff topography will be measured with a device that was developed for ENDS and adapted for JUUL. The software converts signals to airflow (ml/sec) and integrates the flow data, producing measures of puff volume, duration, number, and inter-puff interval (IPI).
7. Plasma nicotine: Plasma nicotine is a standard measure in acute effects lab models for tobacco products. Blood samples (~10 ml) will be drawn via a butterfly needle from the participants' forearm vein before ENDS use session onset and within 10 minutes of its end. Plasma samples will be frozen immediately at −80°C, to be analyzed later by our Forensic Chemistry Lab at FIU using Liquid Chromatography Mass Spectrometry.
8. Aldehydes (playback): Derivatized aldehyde- carbonyl species are extracted from the 2,4-dinitrophenylhydrazine cartridges in 90/10 (vol/vol) ethanol/acetonitrile and quantified by HPLC-UV. We will assess 14 priority aldehyde species that are associated with harm including formaldehyde, acetaldehyde, acetone, acrolein, propionaldehyde, crotonaldehyde, methacrolein, butyraldehyde, benzaldehyde, valeraldehyde, tolualdehyde, hexaldehyde, glyoxal and methylglyoxal.

   Aerosol generation and sampling: The analytical assessments will be conducted at the AUB Aerosol Research Laboratory (ARL). The deidentified puff topography data will be shared via Microsoft Sharepoint between FIU and AUB Aerosol Lab, thus, all communication will be encrypted between client and server using SSL 2048 bit keys. Access to the lab and data will be granted only by permission of Drs. Maziak (PI), and Shihadeh (local PI at AUB). Once the puff topography files have been received by the ARL, we will use ALVIN (Aerosol Lab Vaping Instrument) to draw aerosols from the ENDS devices, for each of the 240 topography sessions. For each session, ALVIN will be programmed to reproduce the puff topography data generated in the clinical lab.

The generated aerosol will be drawn through a Gelman type A/E 47mm glass fiber filter followed by a 2,4-dinitrophenylhydrazine-coated silica cartridge (type LpDNPH H10) cartridge, for nicotine and aldehydes quantification, respectively. The pods (e.g. JUUL) and batteries will be shipped from the same batch as those used at FIU to minimize biases. A new pod will be used for every session. Each pod will be pre-conditioned before sampling by drawing 15 4-second puffs at 1 LPM using ALVIN. We will use a quality assurance protocol and ENDS electrical performance tester developed at the ARL to ensure that each battery and pod are within tolerance.

Chemical analysis: The ARL is equipped with gas chromatographs with a flame ionization detector and a mass spectrometer (GC-FID, GC-MS), as well as and High-performance liquid chromatography-Mass Spectrometry (HPLC-MS) for nicotine and aldehydes analysis. ARL optimized analytical methods have been used in several NIH-funded studies of ENDS nicotine and aldehydes emissions.

9. Three-month follow-up call: Assesses Harm perception, nicotine preference, and changes in ENDS and tobacco use.

**Table 1: Outcome Measures**<br>* Only during baseline	**Measurement time**			
	**Pre**	**During**	**Post**	**3 month**
Demographics*	X			
Tobacco use history	X			X
ENDS Use satisfaction (mCEQ subscale)			X	
Penn State Electronic Cigarette Dependence Index (10 items)	X			
Questionnaire of Smoking Urges (7- point Likert scale)	X		X	
Minnesota Nicotine Withdrawal Scale (score range from 0-100)	X		X	
ENDS Visual analog scale (0 not at all to 100 extremely)			X	
Duke Sensory Scale (7-point Likert scale)			X	
Peer and family influence	X			
Harm perception	X		X	X
Intention to quit or use in the future	X		X	X
Nicotine preference, changes in ENDS use				X
Reasons for ENDS use	X			
Puff topography (puff volume/ml, duration/sec; inter-puff interval/sec)		X		
Cardiovascular measures (BP; HR; oximetry)		X		
Plasma nicotine (ng/ml)	X		X	
Nicotine (playback)			X	
Aldehydes (playback)			X	

Statistical analysis

Topography data will be processed automatically by the topography instrument software to eliminate closely spaced puffs (i.e., IPIs < 300 msec). Such puffs are assumed to be part of the previous puff and the recorded volume and duration values will be added to that preceding puff. After this procedure, any puffs less than 5 ml will be considered an artifact and automatically discarded. The remaining data will be averaged for each participant in each condition using all remaining values for puff volume, duration, number, and IPI. For plasma nicotine, values below the limit of quantitation (LOQ) will be replaced with the value of the LOQ, 2 ng/mL.

Demographic data will be examined and compared to determine if there are significant between- group differences on measures that may be related to study outcome (some differences are expected due to design such as past 30-day ENDS use) though these are expected to be minimized due to randomization. Unexpected between-group differences will be considered as potential adjustment covariates in the primary analysis. We will generate descriptive statistics which include means, standard deviations, frequencies, and proportions depending on the scale of the outcome by NR factor, time and group assignment. Univariate comparisons of the outcomes by the mentioned conditions will consist of repeated measures ANOVA and paired t-test (for 2X2 within factors) and two-sample t-test for between factor, or their non-parametric equivalents (Wilcoxon signed-rank test and Friedman test for within factors and Wilcoxon Mann-Whitney U test for between factor) if the parametric assumptions are not met. Analysis of categorical outcomes will involve a chi-square test of independence for between factors or McNemar’s test for paired proportions. The primary analysis of each outcome will involve analysis of variance with two within-subject factors (nicotine concentration: (5%, 3%) or (0% 5%)) and time (pre-post-use). This approach will be extended into a mixed- effects linear regression model to adjust or study the effects of personal attributes such as sex, education, race, and SES. Furthermore, we will consider clinical/physiologic factors such as respiratory symptoms in a similar modeling approach described above, as a function of experimental factors and demographic covariates, in order to quantify the harm or disease risk. In a case of a non-linear relationship between these factors, we will apply outcome transformations or use non-linear splines to accurately describe these outcomes as a function of mentioned covariates. Given the known sex-related differences in nicotine dependence, to assess if sex has any effect on the results, it will be considered as a covariate and we will test the pre-planned interaction with it to determine the heterogeneity of the study outcomes by sex as well as potential differential effects. Similarly, for categorical outcomes we will apply generalized linear models and estimating equations with the same main effects as described above, modeling within factors as random effects. We will test for the carry- over effect statistically and will control for it when testing for the primary factors. We will also use a one-way ANOVA to test the effect of NR on aldehyde emissions and multiple linear regression analysis to assess the influence of puff topography data (puff volume, duration, number, and interpuff interval) on aldehyde emissions. Additionally, we will measure the relation between the plasma nicotine levels obtained in the clinical lab and measured nicotine yields obtained in the analytical lab using linear regression. Significance levels will be adjusted for violations of the sphericity assumption using Huynh-Feldt corrections. All differences will be considered significant at the alpha level of 0.05 using adjusted p- values where appropriate. Due to the nature of laboratory data collection, missing data is rare and it usually occurs due to instrument malfunction. All data are collected and checked for completeness by laboratory staff. In the case of missing data, which we expect to be minimal, we will use a regression model based on multiple imputations with all available

outcome data, demographic, and clinical variables as predictors. This will be carried out in 25 random repetitions under missing at random assumption, analyzed using methods described above and results will be combined to produce unbiased overall estimates and standard errors of the experimental conditions. As a sensitivity analysis completers results will be compared with multiple imputation results.

Protection of human subjects

All three Aims involve human research subjects that will participate in a crossover lab study that will take place at Maziak's Clinical Research Lab for Tobacco Smoking at Florida International University (FIU; https://stempel.fiu.edu/clinical-research-lab-tobacco-smoking). Prior to initiating the study, the project principal investigator at the study site (Maziak; FIU) will submit and obtain IRB approval. In addition, all IRBs in participating institutions require that all key personnel of studies involving human subjects undergo training on human research subjects' safety (e.g., http://research.fiu.edu/irb/) and receive a certificate from the Collaborative IRB Training Initiative Program (CITI; https://www.citiprogram.org/) prior to any study (part of the IRB approval). A study guide will be developed for the lab study, and the procedures will be followed. All study personnel will be trained in all study procedures, including recruitment, informed consent, and data collection methods. Adherence to the procedures in the study guide will be assured by periodic assessment and retraining. Prior to any data collection, participants will provide written IRB-approved consents that explain the study objectives, procedures, risk and benefits, measures to protect the confidentiality, and voluntary participation/withdrawal from the study at any time. Should changes to the study protocol become necessary, protocol amendments will be submitted to the IRB for approval before implementation. All recruitment materials will be IRB approved.

A proposed change in the study

NIDA Standardized Research Electronic Cigarette (SREC) for Clinical ResearchA proposed change in the study (R01 DA053587, PI: Wasim Maziak):

Brief description of study

Reducing ENDS nicotine levels carries the risk of compensatory puffing and increased exposure. Evidence suggests that such compensation is dose and nicotine dependence related, where very low nicotine products and beginning users are likely to have minimal compensation. Accordingly, assessing the potential role of nicotine reduction regulations to reduce ENDS use and addiction requires standardized comparisons involving a range of nicotine levels and ENDS users. The original study involved recruiting current ENDS users (n=120; 21-35 yrs), for a 3X2 within-subject factorial crossover lab study (3 nicotine conditions; 5%, 3%, 0%) x 2 time; pre-post use). The original plan was to use Juul 5% and 3%, and Juul compatible pods with 0% nicotine as a proxy for the very low nicotine level in the same subjects. Our main hypothesis is that *nicotine reduction will be associated with less satisfaction, withdrawal suppression, and intention to use and that such an effect will be more pronounced in total vs. partial nicotine reduction.*

Brief description of the problem

We examined and compared the puff draw resistance, total particulate mattatter and nicotine emissions from our JUUL-compatible pods (W01) with JUUL-manufactured pods using the AUB lab vaping instrument which was programmed to draw 15 puffs of 4sec duration and 1.5LPM flow rate. We selected nine pods from each product, each removed from a different package and measured the emissions for each, resulting in nine samples per product. We used the same fully charged JUUL battery to power all pods. We found that although both pods are similar in appearance, W01 exhibited significantly lower puff draw resistance (2 times lower) which may influence puff duration and volume in the clinical sessions. We also found that W01 emitted significantly higher TPM and nicotine (2.5 times higher) compared to JUUL-manufactured pods even when powered by the same JUUL battery. These differences may result in differences in user vaping behavior (puff topography parameters), as well as differences in toxicant emissions.

Brief description of the solution

We propose to split this study into 2 parts (studies), whereby the first part will be 2X2 comparing Juul 5% and 3% to test the effects of partial nicotine reduction as was planned, and the second part will be using the NIDA Standardized Research Electronic Cigarette (SREC) for Clinical Research 5% and 0% (placebo), to test the effect of the total reduction using the same pods and juices from the same manufacturer. We believe that this setup will provide us with way more standardized comparisons for this study while still maintaining all the outcomes that we originally were set up to achieve. The only drawback of this arrangement is the potential delay and increased number of required subjects, but we believe that this will be offset by having better retention (less loss to follow up) for a 2 session protocol compared to the 3 sessions originally planned. This change will not affect the budget or require additional funds. previously).

Best

Wasim Maziak, MD, PhD

IRB amendments

FLORIDA INTERNATIONAL UNIVERSITY

Office of Research Integrity
Research Compliance, MARC 414

MEMORANDUM

**To**:	Dr. Wasim Maziak
**CC:**	File
**From:**	Maria Melendez-Vargas, MIBA, Coordinator
**Date:**	June 17, 2022
**Proposal Title:**	"Assessing the effect of nicotine reduction on ENDS user's addiction and exposures"
	**Approval #** IRB-21-0417-AM06
	**Reference #** 110737

The Social and Behavioral Institutional Review Board has approved the following modification(s):

- Update Study split into 2 parts.
- Summary of the Research: Replaced “3x2” with “2x2” to reflect the new study design and described the conditions of the 2 sessions either (3%,5%) or (0%,5%).
- Duration of Participation: Replaced “three sessions” with “two sessions”.
- Updated the telephone eligibility screening to mention that the new number of sessions is “two sessions” instead of “three sessions”.
- Description of Compensation or Incentives and Breakdown of Compensation or Incentives: Replaced “three sessions” with “two sessions” and fixed the compensation by replacing $275 with $200 participants will be attending only 2 sessions ($75 each) and will receive a 3-month follow-up call ($50)).
- Description of Methods & Activities: Replaced “three sessions” with “two sessions” and replaced “three within- subject factors; nicotine concentration (0%, 3%, 5%)” with “two within-subject factors; nicotine concentration (0%, 5%) or (3%, 5%)”.
- Amount of Blood and Frequency: Replaced three sessions with two sessions.
- FDA Approved Products: Replaced three sessions with two sessions and added the NIDA Standardized Research Electronic Cigarette (SREC) for Clinical Research
- Summary of Side Effects, 8.4 Minimizing Risks, Harms, and/or Discomforts, 8.3 Reasonably Expected Risks, Harms, and/or Discomforts: where we replaced "three sessions" by "two sessions"
- Updated the consent forms to reflect the new study design (2 sessions instead of 3 sessions, and

the new incentive).

- Minor changes to Inclusion & Exclusion Criteria added "> 5 times/month in the past year" to clarify the exclusion criteria: "Report regular use of any other tobacco/nicotine product (e.g., e-cig, pipes, cigars)."

There are no additional requirements in regards to your study. However, if there are further changes in the protocol after you commence your study, then you are required to resubmit your proposal for review. As a reminder, you are still require to receive continuing review and re-approval prior to your expiration date **09/28/24.** For further information, you may visit the FIU IRB website at http://research.fiu.edu/irb.

HIPAA Privacy Rule: N/A

Special Conditions: N/A

For further information, you may visit the IRB website at http://research.fiu.edu/irb.

MMV/em

Study Protocol (amendment)

Assessing the effect of nicotine reduction on ENDS users' addiction and exposure

Background

The use of electronic nicotine delivery systems (ENDS) has reached epidemic levels among young people in the US. While estimates vary, ENDS have become the leading tobacco product used by young people in this country. ENDS heat and vaporize a nicotine-containing liquid to produce an inhalable aerosol mist. In addition to dependence-producing nicotine, ENDS emit other toxicants including aldehydes/carbonyls, a class of potent respiratory toxicants implicated in most non-cancer pulmonary diseases in cigarette smokers. Evidence indicates that young people using ENDS are likely to accelerate use, become nicotine dependent (ND), and initiate cigarette smoking. Epidemiological studies and market analysis moreover, suggests that "pod-mod" ENDS, JUUL in particular, had the biggest impact on the ENDS epidemic among young people. Unlike older ENDS generations, JUUL pioneered the use of nicotine salts which allowed to deliver high doses of nicotine in a smooth (protonated) form to users. Thus, addressing the addictiveness of ENDS through nicotine reduction (NR) can be a major regulatory strategy to reduce ENDS use among young people. In cigarette smokers, several observational studies and clinical trials have shown that smokers who switch to low nicotine cigarettes reduce daily nicotine intake, smoke fewer cigarettes, report lower dependence, and show higher trends in quitting. Similar patterns are expected in ENDS users, as our preliminary study of NR in young JUUL users shows that it leads to decreased satisfaction, dependence suppression, and intention to use in the future. We also observed significant differences in puff topography parameters [i.e. mean (SD) of total inhaled volume 2.9 (1.5) vs. 1.6 (0.7) L: $p = 0.03$] consistent with compensatory puffing behavior in response to lower nicotine concentration (3% vs 5%). However, reducing ENDS nicotine levels carries the risk of compensatory puffing and increased exposure. Evidence suggests that such compensation is dose and ND related, where very low nicotine products and beginning users are likely to have minimal compensation. Accordingly, assessing the potential role of NR regulations to reduce ENDS use and addiction requires standardized comparisons involving a range of NR levels and ENDS users at different stages of their use trajectory. It also requires assessing a wide array of outcomes to evaluate the effects of compensatory puffing on exposure to key pulmonary toxicants. These goals can be achieved by applying within-between subject comparisons to assess responses to a range of NR levels (partial vs. total) in ENDS users with different use profiles. Our team has pioneered the use of clinical and analytical lab methods to provide rapid and robust evidence about promising tobacco product manipulations for regulatory purposes (e.g. flavor). We plan to use these methods to compare among ENDS (e.g. JUUL) users the effect of 5% nicotine concentration, 3% (partial NR) or 0% (total or nicotine-free) NR on dependence, satisfaction, puffing behavior, clinical outcomes, and toxicant exposure. Our overarching hypothesis is that NR for ENDS will be associated with less satisfaction, withdrawal suppression, and intention to use and that such an effect will be more pronounced in total vs. partial NR. Our secondary hypothesis is that with very low nicotine ENDS and low dependence users, NR will cause minimal compensatory puffing and increased exposures. We will recruit current ENDS users (n=120; 21-35 yrs), for a 2X2 within- subject factorial crossover lab study. The nicotine conditions (5%, 3%) or (0%, 5%) x 2 time (pre-post) are the within-subject factors. A follow-up call at 3-months post-lab will be made to assess nicotine preference and changes in ENDS use.

Study Aims

Aim 1: To assess puffing behavior in response to NR on ENDS users. Under the same protocol, puff topography (e.g. puff volume, puff frequency, duration) will be assessed continuously during the 2 sessions per study, while plasma nicotine will be measured pre-post sessions to assess nicotine boost and its correlation to puffing parameters. This aim will help understand users' compensatory puffing in response to NR and its effect on plasma nicotine.

Aim 2. To assess exposure to toxicants associated with NR among ENDS users. We will measure toxicant emissions (14 aldehydes) in the analytical lab using a smoking robot that

reproduces the puff- by-puff behavior (playback) of each participant for both nicotine conditions per study. This aim will reveal how compensatory puffing behavior in response to NR influences acute exposure to pulmonary toxicants.

Aim 3: To assess the subjective and clinical effects of NR on ENDS users. This study will focus on pre-post-use assessment of craving, withdrawal, satisfaction, intention to quit or use in the future, and clinical symptoms (e.g. dry mouth, eye irritation, palpitation, nausea). This aim will show ENDS users' response to NR on product satisfaction, dependence, and clinical symptoms.

The proposed studies will give clear evidence on the potential of NR regulations to limit the addictiveness and use of ENDS, and to help predict any potential side effects of NR on ENDS users.

Study Design

This study is divided into 2 parts (studies), whereby the first part will be 2X2 comparing JUUL/NJOY 5% and 3% to test the effects of partial nicotine reduction, and the second part will be using the NIDA Standardized Research Electronic Cigarette (SREC) for Clinical Research 5% and 0% (placebo), to test the effect of the total reduction using the same pods and juices from the same manufacturer.

Sample size

All power calculations were performed with PASS19 (PASS, 2019). We built our calculation of the sample size for this study based on our preliminary study of the effect of NR on subjective (e.g. craving, urges) and puff topography measures (pls. see Preliminary Studies), where medium effect size (Cohen's f=0.2) depending on nicotine condition was detected. Expecting a maximum 20% loss due to noncompliance with abstinence or study protocol (Vargas-Rivera et al., 2020), this study will include a dropout inflated sample of 150 ENDS users. Using repeated measures ANOVA F-test with 2 within and 1 between factors, a total dropout adjusted sample size of 120 (2X60) participants will have at least 80% power to detect small to medium-size effect (Cohen's f=0.1-0.2) or larger for 2 within-subject (NR condition and time) and 1 between subject (use frequency) factors, as well as their interactions at 0.05 level of significance, assuming sphericity and a moderate correlation (0.5) among repeated measures (Cohen, 1988).

Participants and recruitment

Participants: We will recruit 120 current ENDS users defined as using ENDS either daily or occasionally in the past 30 days (as in Vargas-Rivera et al., 2020). Individuals who report smoking cigarettes or other tobacco/nicotine products will be included to increase the generalizability of results and enhance recruitment provided that they don't use these tobacco products >5 times/past month (as in Ben Taleb et al., 2020). As our study focuses on nicotine dependence related parameters, any substantial use of other tobacco/nicotine products will likely bias our planned assessments. Similarly, the pre-study 12 hours abstinence period from all tobacco/nicotine products and using the same product type/brand ensures that study outcomes reflect mainly each session's condition. We aim to recruit equal numbers of men and women.

Inclusion and exclusion criteria: Participants need to be generally healthy, between 21-35 years old, provide written informed consent, and agree to attend the lab on two occasions and use their ENDS product according to the study protocol. Participants will be asked to abstain from ENDS and all tobacco/nicotine products use for at least 12 hours prior to each session. The abstinence period is needed to clear nicotine from prior-to-study use and ensure that all study measures are influenced mostly by the study conditions. Testing short-term abstinence

of ENDS, which does not produce carbon monoxide (CO) is an ongoing challenge (Hiler et al., 2017). Therefore, we will follow published recommendations (Blank et al., 2016) to use the gold standard of plasma nicotine to eliminate data from non-compliant participants later. Because this will entail a loss of data, we will try to minimize that and improve compliance by asking the participants to arrive at the scheduled study sessions one hour early for an observation period, and complete a CO test (Blank et al., 2016). The CO test will be good to detect combustible tobacco products' use (e.g. cigarette smoking), and act as a bogus pipeline for ENDS use (Blank et al., 2016). This procedure has been shown to cut participants' non-adherence to the abstinence condition to <10% (Spindle et al., 2018), which is consistent with our own experience (Vargas-Rivera et al., 2020). A blood draw will be completed before each session, abstinence will be confirmed using plasma nicotine levels less than 5 ng/ml (Benowitz, Hukkanen, & Jacob, 2009). Individuals with a self-reported history of chronic disease, psychiatric conditions, history of cardiovascular disease, low or high blood pressure (BP) (systolic BP>150 mm Hg, or diastolic BP>100 mm Hg), seizures, or regular use of prescription medications (other than vitamins or birth control) will be excluded (St Helen et al., 2017). This will be assessed by history and physical examination conducted by the research nurse during a screening visit. The research nurse will have access to an on-call Medical Monitor (physician) in case of concern or for consultation at any point during the study (as is the current practice in our lab). The physical examination will include measuring BP, heart rate (HR), temperature, pulse oximetry, and weight/height. Importantly, considering the current COVID-19 pandemic we will follow CDC and FDA guidelines for clinical research and COVID-19 to minimize any potential risk of infection transmission as detailed in our Human Subject Protection part (CDC, 2020b; FDA, 2020c).

Briefly, we will apply a protocol consisting of 5 main areas;
1-Screening of Participants for COVID-19;
2- Social and Physical Distancing;
3- Use of Personal Protective Equipment (PPE);
4- Cleaning and disinfecting;
5- Personal Hygiene and Hand Washing.

Participants with symptoms/history suggestive of COVID-19 (e.g. □100.4°F, cough, contact) or with a history of cannabis use (risk for e-cigarette or vaping product use-associated lung injury-EVALI) will be excluded from participation (details in Human Subjects Protection). As knowledge about COVID-19 is rapidly evolving we will apply best practices at the time of study to protect human subjects and our staff (e.g. require vaccination record if such becomes feasible). FIU has started providing vaccination to all research staff with direct contact with human subjects, and free-of-charge COVID-19 testing facilities have become available within and in the vicinity of FIU (e.g.FIU-Curative., 2020). Women will be excluded if they are breast-feeding or test positive for pregnancy (by urine pregnancy testing) at screening (St Helen et al., 2017). Finally, we will also exclude individuals if they intend to quit e-cigarette within the next 3 month and if they use THC in e-cigarette.

Recruitment: We will recruit participants using a combination of online, offline, and in-person recruitment methods to ensure successful recruitment (Buller et al., 2012). Offline items such as posters, flyers, and study inserts will be posted and distributed on FIU and other university campuses in Miami (e.g. University of Miami), nearby off-campus locations, and around ENDS shops in Miami. Online recruitment will include student listservs, Facebook, and other social media. In-person recruitment will involve handing out flyers and word-of-mouth targeting vape shops in Miami. Recruitment materials will be Institutional Review Board (IRB) approved and will have contact information (phone, e-mail) for initial eligibility screening. Based on previous experience, we expect to study 10 subjects/month (Vargas-Rivera et al., 2020). We will continue recruitment until we reach the target sample (120 with complete sessions). While recruitment is always a challenge, Maziak's lab has easily recruited JUUL users in the past year with negligible drop out for 2 session protocol using comparable incentives, to the extent that we had to turn down many eligible participants due to sampling saturation (Vargas- Rivera

et al., 2020). This is due to the widespread use of ENDS, and JUUL among young adults, especially in a large university with about 57,000 students such as FIU, where our lab is located. Participants completing the on-site screening and 2 lab sessions, and 3-month phone follow-up will be compensated a total $200 at the end of the study to encourage completeness.

Screening, and informed consent: Individuals who are identified as potentially eligible based on the phone screening will be asked to attend an onsite-screening, which will involve assessment of inclusion/exclusion criteria, physical examination, COVID-19 screening, and urine pregnancy testing for women. Prior to screening, potential participants will undergo a review of the study and consent procedures to ensure that they understand the study, its risks/benefits, and their rights as research participants. Consented (written) individuals will then undergo a physical examination conducted by the research nurse, who will have access to an on-call Medical Monitor (physician) in case of concern or for consultation at any point of the study as it is the standard practice in our lab. Successfully consented participants will be scheduled for their 1st session and asked to abstain from all tobacco/nicotine use 12 hours prior to it.

Experimental protocol

Participants will be randomly assigned to one of the nicotine concentration conditions (for both parts of the studies, 5% vs. 3% and 5% vs 0%) and attend the lab for two, ~2-h sessions, separated by a 48-h washout period, and differ by nicotine concentration (3%,5%) or (0%, 5%). Session order will be counterbalanced to mitigate order and carry-over effects. At the beginning of the 1st session, participants will provide consent, complete demographic and personal information including age, sex, socioeconomic status, race/ethnicity, and detailed tobacco/nicotine use history. Participants will get familiarized with the study procedures, measures, and they will be given time to adapt to the lab. After adaptation, continuous measurement of physiological responses begins, and 10 ml of venous blood is sampled, lung function tests will be conducted, and participants are asked to respond to subjective measures (pls. see below Outcome Measures). Participants will inhale on their session ENDS ad libitum for up to 60 minutes (as in Vargas-Rivera et al., 2020). At the end of the ENDS use period 10 ml of blood will be sampled, lung function tests repeated, and subjective measures assessed. The session will terminate 30 minutes after the last puff, and the 2nd session is scheduled. A follow-up phone call at 3-month post-study will be made to evaluate nicotine preferences and changes in ENDS and tobacco use.

Outcome measures

We will utilize our clinical lab model to study the effect of NR on the following outcomes assessed according to the plan described in Table 1. All assessments will be based on instruments that are standard in clinical lab studies of addictive behaviors modified for the ENDS. Participants will use a tablet to respond to these measures using RedCap. Briefly, we will assess;

1. Demographic and baseline characteristics; including age, race, sex, and reasons for ENDS use. Other characteristics will include the frequency of ENDS use, and the history of other tobacco/nicotine product use
2. Dependence measures include; 1) Use satisfaction (Modified Cigarette Evaluation Questionnaire (mCEQ); 2) Dependence (Questionnaire of Smoking Urges-brief and the Minnesota Nicotine Withdrawal Scale, Penn State Electronic Cigarette Dependence Index; 3) Puff sensory effects (Duke Sensory Scale).
3. Use related measures including 1) Harm perception 2) Intention to quit or use in the future; 3) Reasons for ENDS use, and 4) Visual Analogue Scale (VAS) to measure ENDS use experience such as pleasantness, enjoyment, and pleasure from use.
4. Clinical symptoms, such as (dry mouth, eye irritation, palpitations, and nausea) will be assessed pre-post ENDS use using standard clinical assessments. Symptoms like cough,

sore throat will be assessed after-session since our participants will be free of these symptoms pre-session to exclude potential COVID-19 cases (pls. see Inclusion and Exclusion criteria).

5. Cardiovascular measures: To monitor participants' vital signs, physiological measures will be monitored during each session such as heart rate, blood pressure, and pulse oximetry using the Noninvasive Patient Monitor 507E, Criticare Systems, Waukesha, WI.
6. Puff topography: Puff topography will be measured with a device that was developed for ENDS and adapted for JUUL. The software converts signals to airflow (ml/sec) and integrates the flow data, producing measures of puff volume, duration, number, and inter-puff interval (IPI).
7. Plasma nicotine: Plasma nicotine is a standard measure in acute effects lab models for tobacco products. Blood samples (~10 ml) will be drawn via a butterfly needle from the participants' forearm vein before ENDS use session onset and within 10 minutes of its end. Plasma samples will be frozen immediately at −80°C, to be analyzed later by our Forensic Chemistry Lab at FIU using Liquid Chromatography Mass Spectrometry.
8. Aldehydes (playback): Derivatized aldehyde- carbonyl species are extracted from the 2,4-dinitrophenylhydrazine cartridges in 90/10 (vol/vol) ethanol/acetonitrile and quantified by HPLC-UV. We will assess 14 priority aldehyde species that are associated with harm including formaldehyde, acetaldehyde, acetone, acrolein, propionaldehyde, crotonaldehyde, methacrolein, butyraldehyde, benzaldehyde, valeraldehyde, tolualdehyde, hexaldehyde, glyoxal and methylglyoxal.

   Aerosol generation and sampling: The analytical assessments will be conducted at the AUB Aerosol Research Laboratory (ARL). The deidentified puff topography data will be shared via Microsoft Sharepoint between FIU and AUB Aerosol Lab, thus, all communication will be encrypted between client and server using SSL 2048 bit keys. Access to the lab and data will be granted only by permission of Drs. Maziak (PI), and Shihadeh (local PI at AUB). Once the puff topography files have been received by the ARL, we will use ALVIN (Aerosol Lab Vaping Instrument) to draw aerosols from the ENDS devices, for each of the 240 topography sessions. For each session, ALVIN will be programmed to reproduce the puff topography data generated in the clinical lab. The generated aerosol will be drawn through a Gelman type A/E 47mm glass fiber filter followed by a 2,4-dinitrophenylhydrazine-coated silica cartridge (type LpDNPH H10) cartridge, for nicotine and aldehydes quantification, respectively. The pods (e.g. JUUL) and batteries will be shipped from the same batch as those used at FIU to minimize biases. A new pod will be used for every session. Each pod will be pre-conditioned before sampling by drawing 15 4-second puffs at 1 LPM using ALVIN. We will use a quality assurance protocol and ENDS electrical performance tester developed at the ARL to ensure that each battery and pod are within tolerance.

   Chemical analysis: The ARL is equipped with gas chromatographs with a flame ionization detector and a mass spectrometer (GC-FID, GC-MS), as well as and High-performance liquid chromatography-Mass Spectrometry (HPLC-MS) for nicotine and aldehydes analysis. ARL optimized analytical methods have been used in several NIH-funded studies of ENDS nicotine and aldehydes emissions.
9. Three-month follow-up call: Assesses Harm perception, nicotine preference, and changes in ENDS and tobacco use

**Table 1: Outcome Measures**<br>* Only during baseline	**Measurement time**			
	**Pre**	**During**	**Post**	**3 month**
Demographics*	X			
Tobacco use history	X			X
ENDS Use satisfaction (mCEQ subscale)			X	
Penn State Electronic Cigarette Dependence Index (10 items)	X			
Questionnaire of Smoking Urges (7- point Likert scale)	X		X	
Minnesota Nicotine Withdrawal Scale (score range from 0-100)	X		X	
ENDS Visual analog scale (0 not at all to 100 extremely)			X	
Duke Sensory Scale (7-point Likert scale)			X	
Peer and family influence	X			
Harm perception	X		X	X
Intention to quit or use in the future	X		X	X
Nicotine preference, changes in ENDS use				X
Reasons for ENDS use	X			
Puff topography (puff volume/ml, duration/sec; inter-puff interval/sec)		X		
Cardiovascular measures (BP; HR; oximetry)		X		
Plasma nicotine (ng/ml)	X		X	
Nicotine (playback)			X	
Aldehydes (playback)			X	

Statistical analysis

Topography data will be processed automatically by the topography instrument software to eliminate closely spaced puffs (i.e., IPIs < 300 msec). Such puffs are assumed to be part of the previous puff and the recorded volume and duration values will be added to that preceding puff. After this procedure, any puffs less than 5 ml will be considered an artifact and automatically discarded. The remaining data will be averaged for each participant in each condition using all remaining values for puff volume, duration, number, and IPI. For plasma nicotine, values below the limit of quantitation (LOQ) will be replaced with the value of the LOQ, 2 ng/mL.

Demographic data will be examined and compared to determine if there are significant between- group differences on measures that may be related to study outcome (some differences are expected due to design such as past 30-day ENDS use) though these are expected to be minimized due to randomization. Unexpected between-group differences will be considered as potential adjustment covariates in the primary analysis. We will generate descriptive statistics which include means, standard deviations, frequencies, and proportions depending on the scale of the outcome by NR factor, time and group assignment. Univariate comparisons of the outcomes by the mentioned conditions will consist of repeated measures ANOVA and paired t-test (for 2X2 within factors) and two-sample t-test for between factor, or their non-parametric equivalents (Wilcoxon signed-rank test and Friedman test for within factors and Wilcoxon Mann-Whitney U test for between factor) if the parametric assumptions are not met. Analysis of categorical outcomes will involve a chi-square test of independence for between factors or McNemar's test for paired proportions. The primary analysis of each outcome will involve analysis of variance with two within-subject factors (nicotine concentration: (5%, 3%) or (0% 5%)) and time (pre-post-use). This approach will be extended into a mixed- effects linear regression model to adjust or study the effects of personal attributes such as sex, education, race, and SES. Furthermore, we will consider clinical/physiologic factors such as respiratory symptoms in a similar modeling approach described above, as a function of experimental factors and demographic covariates, in order to quantify the harm or disease risk. In a case of a non-linear relationship between these factors, we will apply outcome transformations or use non-linear splines to accurately describe these outcomes as a function of mentioned covariates. Given the known sex-related differences in nicotine dependence, to assess if sex has any effect on the results, it will be considered as a covariate and we will test the pre-planned interaction with it to determine the heterogeneity of the study outcomes by sex as well as potential differential effects. Similarly, for categorical outcomes we will apply generalized linear models and estimating equations with the same main effects as described above, modeling within factors as random effects. We will test for the carry- over effect statistically and will control for it when testing for the primary factors. We will also use a one-way ANOVA to test the effect of NR on aldehyde emissions and multiple linear regression analysis to assess the influence of puff topography data (puff volume, duration, number, and interpuff interval) on aldehyde emissions. Additionally, we will measure the relation between the plasma nicotine levels obtained in the clinical lab and measured nicotine yields obtained in the analytical lab using linear regression. Significance levels will be adjusted for violations of the sphericity assumption using Huynh-Feldt corrections. All differences will be considered significant at the alpha level of 0.05 using adjusted p- values where appropriate. Due to the nature of laboratory data collection, missing data is rare and it usually occurs due to instrument malfunction. All data are collected and checked for completeness by laboratory staff. In the case of missing data, which we expect to be minimal, we will use a regression model based on multiple imputations with all available outcome data, demographic, and clinical variables as predictors. This will be carried out in 25

random repetitions under missing at random assumption, analyzed using methods described above and results will be combined to produce unbiased overall estimates and standard errors of the experimental conditions. As a sensitivity analysis completers results will be compared with multiple imputation results.

Protection of human subjects

All three Aims involve human research subjects that will participate in a crossover lab study that will take place at Maziak's Clinical Research Lab for Tobacco Smoking at Florida International University (FIU; https://stempel.fiu.edu/clinical-research-lab-tobacco-smoking). Prior to initiating the study, the project principal investigator at the study site (Maziak; FIU) will submit and obtain IRB approval. In addition, all IRBs in participating institutions require that all key personnel of studies involving human subjects undergo training on human research subjects' safety (e.g., http://research.fiu.edu/irb/) and receive a certificate from the Collaborative IRB Training Initiative Program (CITI; https://www.citiprogram.org/) prior to any study (part of the IRB approval). A study guide will be developed for the lab study, and the procedures will be followed. All study personnel will be trained in all study procedures, including recruitment, informed consent, and data collection methods. Adherence to the procedures in the study guide will be assured by periodic assessment and retraining. Prior to any data collection, participants will provide written IRB-approved consents that explain the study objectives, procedures, risk and benefits, measures to protect the confidentiality, and voluntary participation/withdrawal from the study at any time. Should changes to the study protocol become necessary, protocol amendments will be submitted to the IRB for approval before implementation. All recruitment materials will be IRB approved.
