# Supplementary material for: Partial Nicotine Reduction and E-Cigarette Users’ Puffing Behaviors Among Adults Aged 21 to 35 Years: A Randomized Crossover Clinical Trial
Source: JAMA Netw Open. 2024 Jul 26;7(7):e2422954. doi: 10.1001/jamanetworkopen.2024.22954 (PMC11282440; doi:10.1001/jamanetworkopen.2024.22954)
Supplement: Supplement 2. — eMethods eFigure 1. QQ Plot to Assess the Normality of All Outcomes in Table 2 and Table 3 by Each Session Group (3% vs 5%): Shown Only for Topographic Outcomes eFigure 2. QQ Plot to Assess the Normality of Residuals for All Outcome Variables (Untransformed vs. Log-Transformed): Shown Only for Models With “Nicotine Dependence Levels” as the Independent Variable eTable 1. E-Cigarette Users’ Topography Measured by Nicotine Concentration Session Orders (Order 1 Started With 3%, and Order 2 Started With 5%) (n = 100): Shown Only for Topographic Outcomes eTable 2. Test for Normality and Heteroscedasticity for Model Residuals and Multicollinearity for Variables (Untransformed and Log-Transformed Outcome Variables): Shown Only for Adjusted Models With “Nicotine Dependence Levels” As the Independent Variable eReferences [file jamanetwopen-e2422954-s002.pdf]

Supplemental Online Content

Ferdous T, Roy S, Chowdhury S, et al. Partial nicotine reduction and e-cigarette users' puffing behaviors among adults aged 21 to 35 years: a randomized crossover clinical trial. *JAMA Netw Open*. 2024;7(7):e2422954.
doi:10.1001/jamanetworkopen.2024.22954

eMethods
eFigure 1. QQ Plot to Assess the Normality of All Outcomes in Table 2 and Table 3 by Each Session Group (3% vs. 5%): Shown Only for Topographic Outcomes
eFigure 2. QQ Plot to Assess the Normality of Residuals for All Outcome Variables (Untransformed vs Log-Transformed): Shown Only for Models With "Nicotine Dependence Levels" as the Independent Variable
eTable 1. E-Cigarette Users' Topography Measured by Nicotine Concentration Session Orders (Order 1 Started With 3%, and Order 2 Started With 5%) (n = 100): Shown Only for Topographic Outcomes
eTable 2. Test for Normality and Heteroscedasticity for Model Residuals and Multicollinearity for Variables (Untransformed and Log-Transformed Outcome Variables): Shown Only for Adjusted Models With "Nicotine Dependence Levels" As the Independent Variable
eReferences

This supplemental material has been provided by the authors to give readers additional information about their work.

© 2024 Ferdous T et al. *JAMA Network Open*.

eMethods

Randomization Procedures

Participants were randomly assigned to the order of conditions, receiving either the partially reduced nicotine concentration e-cigarette (condition 1 with 3% nicotine concentration pod) or the regular preferred nicotine concentration e-cigarette (condition 2 with 5% nicotine concentration pod) for their first session of the study. Randomization was stratified by gender, device brand, and nicotine conditions, so there was a balance in order of conditions between males and females, JUUL and NJOY e-cigarette device brands, and 3% and 5% e-cigarette pod conditions (e.g., there were eight groups: male 3% JUUL, female 5% NJOY). Randomization was performed by a Ph.D. research graduate assistant using a SAS random number generator with a uniform distribution following a blocked randomization schema for each of the gender-nicotine condition stratum (blocks of eight for male strata and eight for female strata to maintain equality in sex of participants). Participants were enrolled by the lab manager, and a Ph.D. graduate assistant assigned them to interventions (3% or 5%). A total of 10 participants dropped out (lost to follow-up) before the second visit for both randomization orders (i.e., 4 participants for condition 1→ condition 2, and 6 participants for condition 2→ condition 1) (CONSORT diagram).

Plasma nicotine analysis

Sample Preparation of Plasma Matrix

Sample preparation consisted of thawing and vortexing the sample for 1 minute. A 200 µL aliquot of the sample was then transferred to a plastic Eppendorf tube and 500 µL of cold (-20ºC) acetonitrile was added for protein precipitation. The mixture was vortexed and centrifuged for 10 minutes at ~15000 rpm at room temperature (~20ºC). An aliquot of supernatant was transferred to an LC/MS vial with a conical glass insert. The sample was spiked with nicotine-d4 and cotinine-d3 as the internal standards.

LC-QQQ-MS Analysis Parameters

All samples were analyzed using an Agilent 1290/6460 triple quadrupole LC-MS/MS with Jet Stream electrospray ionization (ESI). Chromatographic separation was accomplished using an Agilent Zorbax Rapid Resolution HD Eclipse Plus C-18 column (3.0 x 50 mm, 1.8 µm). Dynamic multiple reaction monitoring (dMRM) was used to analyze transitions for each analyte and internal standard. Mobile phase A consisted of 5 mM ammonium formate in water with 0.1% formic acid and mobile phase B consisted of acetonitrile with 0.1% formic acid. The elution gradient used was 5% B, held for 1 min, to 95% from 1 to 6 min, at a flow rate of 0.5 mL/min.

The mass spectrometer parameters were as follows: gas temperature of 350 degrees Celsius; a drying gas flow of 5 L/min; nebulizer gas at 45 psi; a sheath gas temperature of 350 degrees Celsius at a flow of 11 L/min; the nozzle voltage was set to 1500 V with capillary voltage set to 4000 V. All data was analyzed on Agilent MassHunter Quantitative software version B8.0.

Sample size calculation

The following sample size calculation is presented from the protocol, which was calculated for the larger study.

All power calculations were performed with PASS19.[1] We built our calculation of the sample size for this study based on our preliminary study of the effect of NR on subjective (e.g. craving, urges) and puff topography measures (pls. see Preliminary Studies), where medium effect size (Cohen's f=0.2) depending on nicotine condition was detected. Expecting a maximum 20% loss due to noncompliance with abstinence or study protocol,[2] this study will include a dropout inflated sample of 150 ENDS users divided into 2 groups of high and low-frequency users. Using repeated measures ANOVA F-test with 2 within and 1 between factors, a total dropout adjusted sample size of 120 (2X60) participants will have at least 80% power to detect small to medium-size effect (Cohen's f=0.1-0.2) or larger for 2 within-subject (NR condition and time) and 1 between subject (use frequency) factors, as well as their interactions at 0.05 level of significance, assuming sphericity and a moderate correlation (0.5) among repeated measures.[3]

For the current study, we have considered the amended protocol (see protocol amendments 1 and 2) and provided the post-hoc sample size calculation accordingly, as given in the main manuscript.

© 2024 Ferdous T et al. *JAMA Network Open*.

Statistical analysis

Model assumptions were checked using a QQ plot to assess the normality of residuals (confirmed by the Shapiro test) and Breusch–Pagan Test to assess heteroscedasticity (eTable 2, Supplement 2). Residuals from the models using untransformed outcome data were not normally distributed, so all outcomes were log-transformed for the final adjusted analysis models, which significantly improved model fit (eFigure 2, Supplement 2). For each model, we assessed multicollinearity using the variance inflation factor (VIF), where a VIF greater than 10 may suggest a concerning correlation between the explanatory variables (eTable 2, Supplement 2).

© 2024 Ferdous T et al. *JAMA Network Open*.

eFigure 1. QQ Plot to Assess the Normality of All Outcomes in Table 2 and Table 3 by Each Session Group (3% vs. 5%): Shown Only for Topographic Outcomes

Vaping session time (3% session)

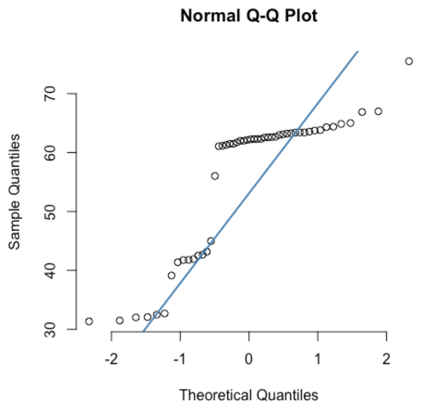

vaping session time (5% session)

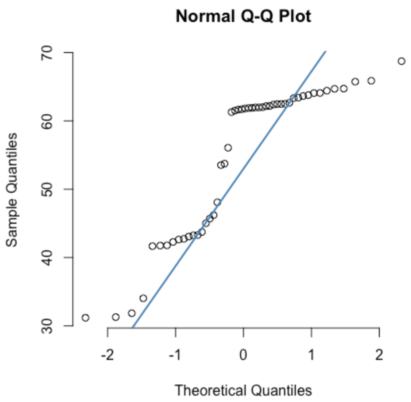

Puffing time (3% session)

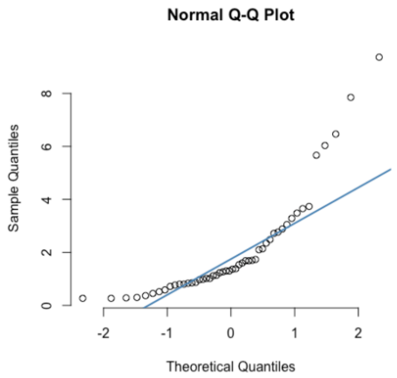

Puffing time (5% session)

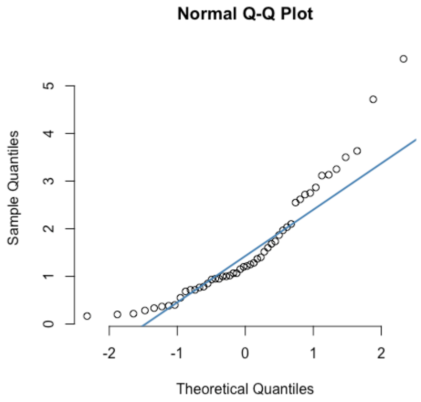

Puff duration (3% session)

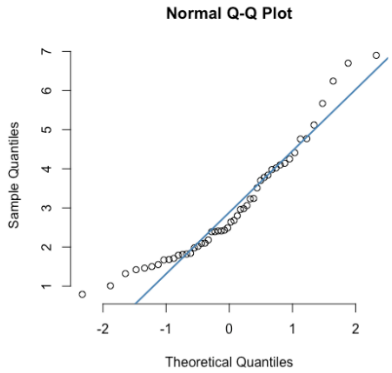

Puff duration (5% session)

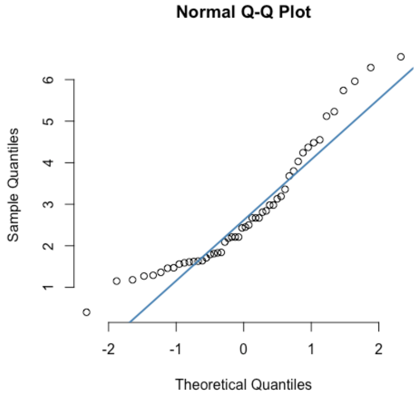

© 2024 Ferdous T et al. *JAMA Network Open*.

Average flow rate (3% session)

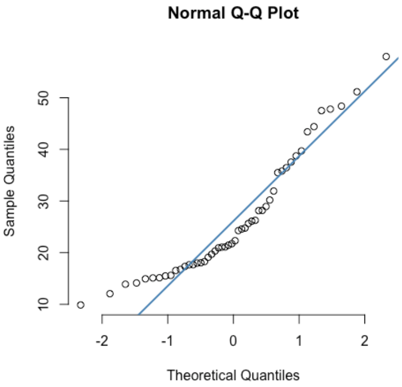

Average flow rate (5% session)

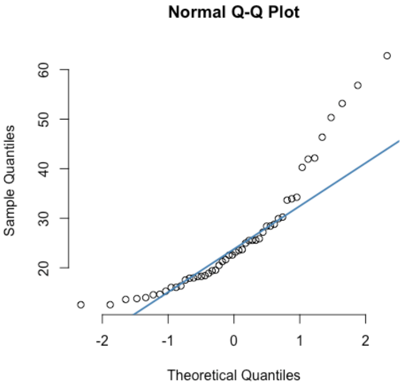

Inter-puff-interval (3% session)

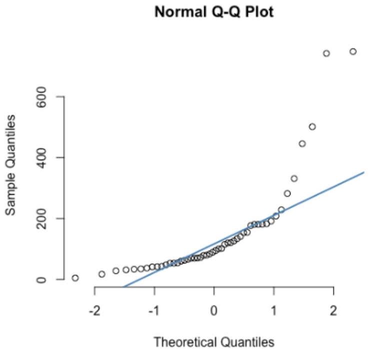

Inter-puff-interval (5% session)

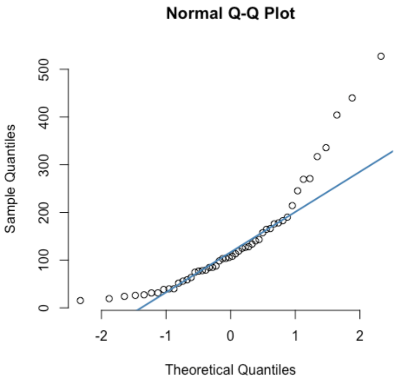

Total puffs (3% session)

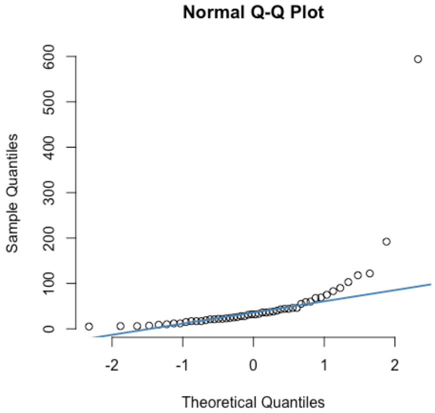

Total puffs (5% session)

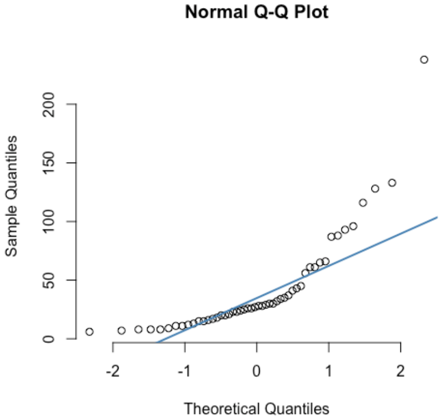

© 2024 Ferdous T et al. *JAMA Network Open*.

Inhaled volume (3% session)

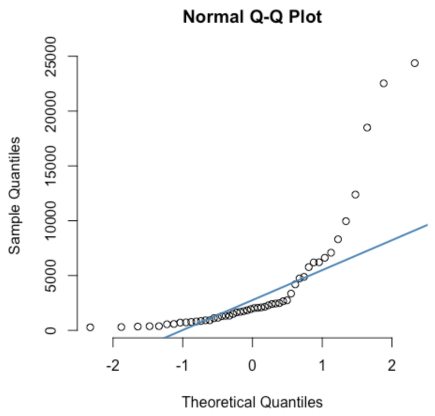

Inhaled volume (5% session)

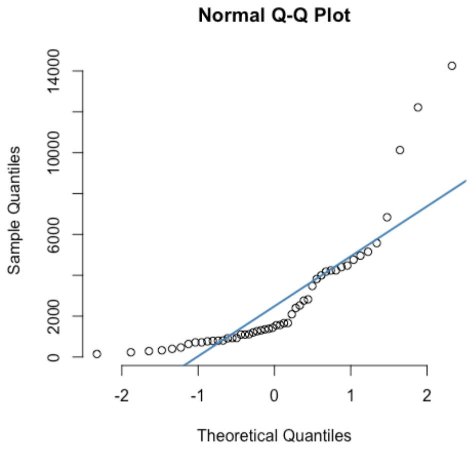

Average puff volume (3% session)

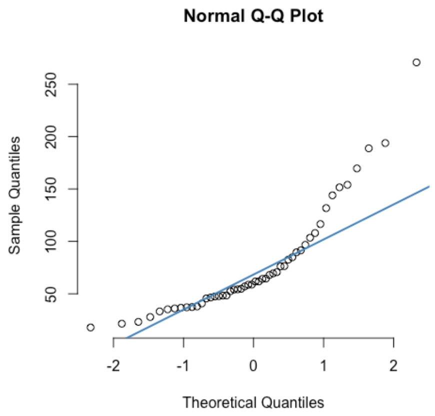

Average puff volume (5% session)

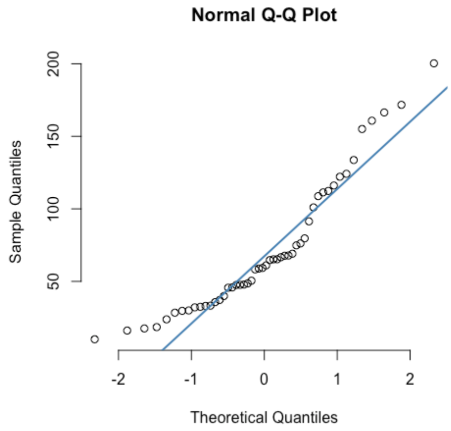

Maximum puff volume (3% session)

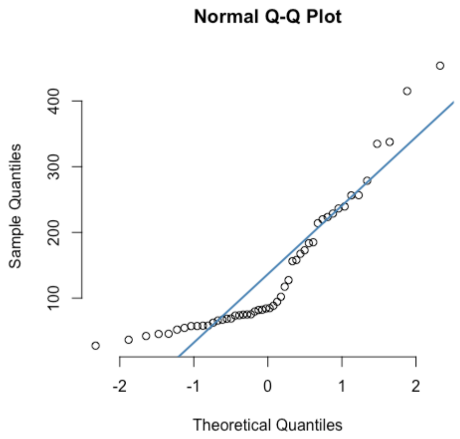

Maximum puff volume (5% session)

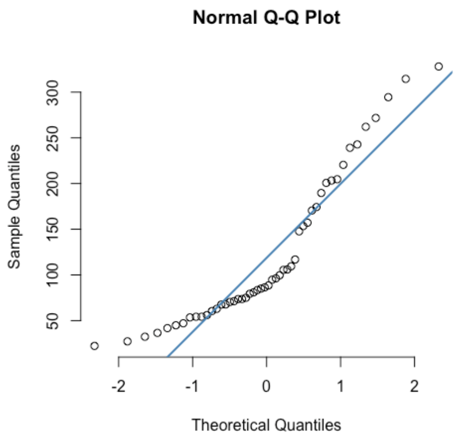

© 2024 Ferdous T et al. *JAMA Network Open*.

eFigure 2. QQ Plot to Assess the Normality of Residuals for All Outcome Variables (Untransformed vs Log-Transformed): Shown Only for Models With "Nicotine Dependence Levels" as the Independent Variable

Puffing time

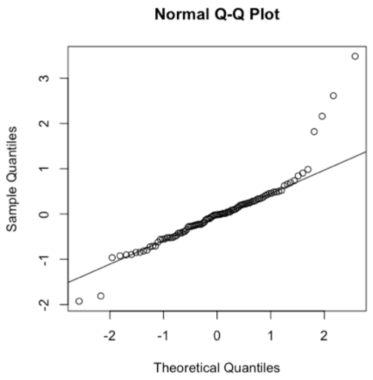

Puffing time (log-transformed)

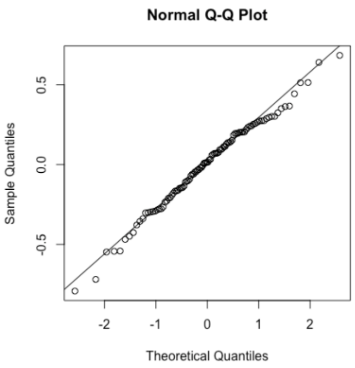

Puff duration

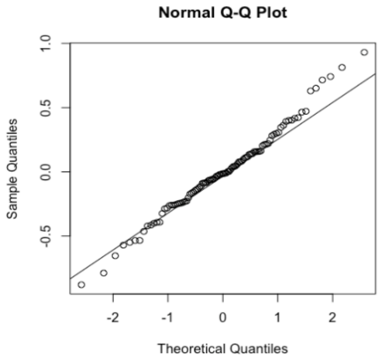

Puff duration (log-transformed)

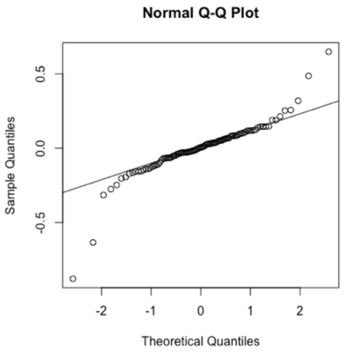

Average flow rate

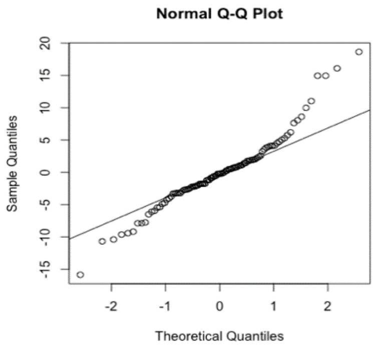

Average flow rate (log-transformed)

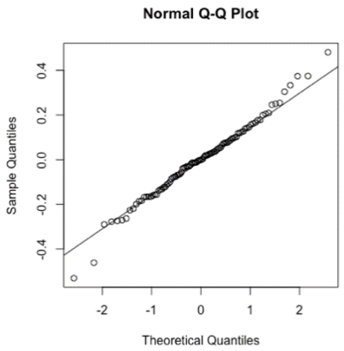

© 2024 Ferdous T et al. *JAMA Network Open*.

Inter-puff-interval

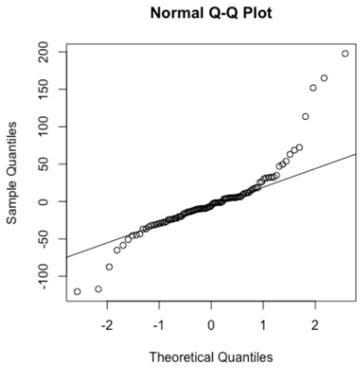

(log-transformed)

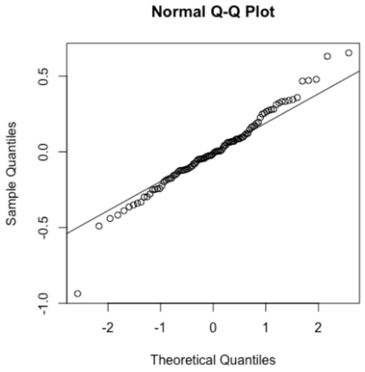

Total puffs

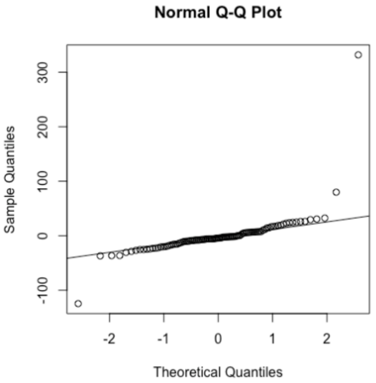

Total puffs (log-transformed)

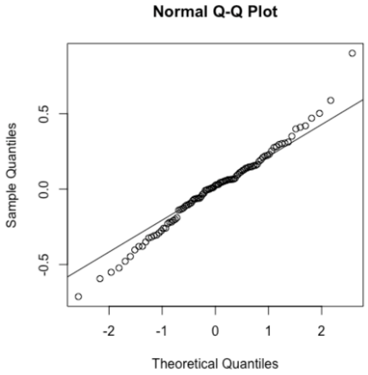

Inhaled volume

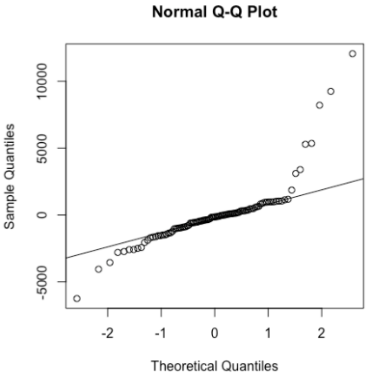

Inhaled volume (log-transformed)

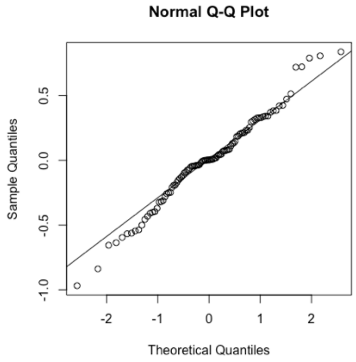

© 2024 Ferdous T et al. *JAMA Network Open*.

Average puff volume

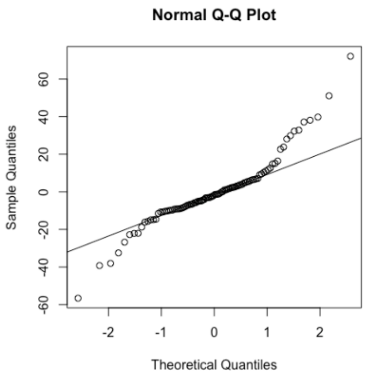

Average puff volume (log-transformed)

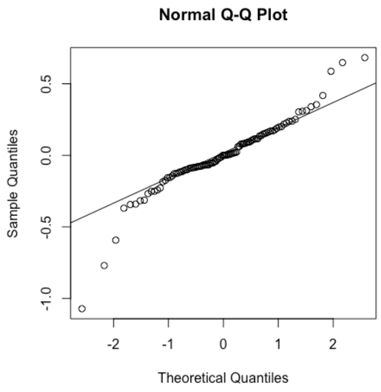

Maximum puff volume

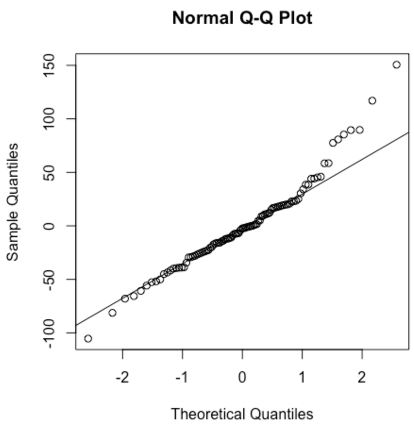

Maximum puff volume (log-transformed)

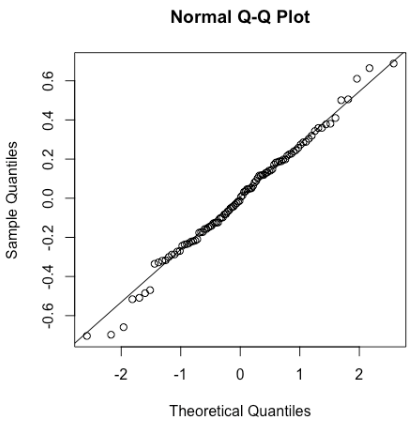

Nicotine-boost

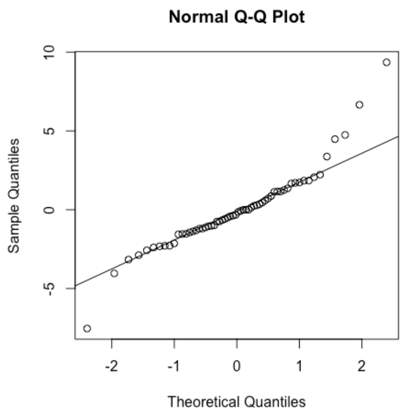

(log-transformed)

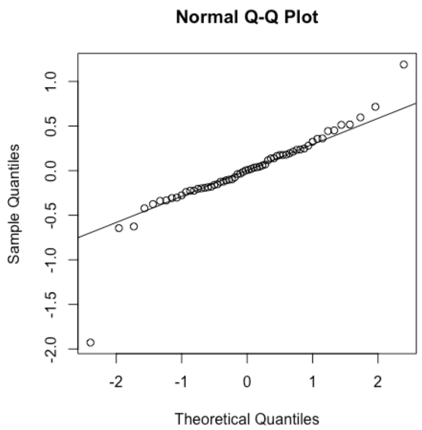

© 2024 Ferdous T et al. *JAMA Network Open*.

eTable 1. E-Cigarette Users' Topography Measured by Nicotine Concentration Session Orders (Order 1 Started With 3%, and Order 2 Started With 5%) (n = 100): Shown Only for Topographic Outcomes

**Topography parameters**	**Wilcoxon rank-sum test** between two between-subject conditions (order of study):<br><br>[Sessions with order 1 (n=51) started with 3% nicotine concentration and order 2 (n=49) with 5% nicotine concentration]	
	**Co-efficient**	**p-value (alpha = 0.05)**
Vaping session time, min	-0.1028611	0.45
Puffing time, min	-0.2450113	0.93
Average Puff duration, sec	0.4510084	0.090
Average flow rate, ml/sec	0.5931533	0.49
Average inter-puff interval, sec	-3.808415	0.52
Number of puffs	-22.2581	0.69
Total inhaled volume, ml	-294.2568	0.73
Average Puff volume, ml	19.16344	0.11
Maximum puff volume, ml	18.30132	0.5014

Normality was assessed using the QQ plot for all outcomes by each group order of sessions, and distributions were not normal.

© 2024 Ferdous T et al. *JAMA Network Open*.

eTable 2. Test for Normality and Heteroscedasticity for Model Residuals and Multicollinearity for Variables (Untransformed and Log-Transformed Outcome Variables): Shown Only for Adjusted Models With "Nicotine Dependence Levels" As the Independent Variable

**Topography parameters**	**Test for normality of residual (Shapiro-Wilk Test)**		**Test for heteroscedasticity of residual (Breusch–Pagan Test)**		**Test for multicollinearity among independent variables using the variance inflation factor (VIF)**	
	**Untransformed**	**log-transformed**	**Untransformed**	**log-transformed**	**Untransformed**	**log-transformed**
	**P-value**	**P-value**	**P-value**	**P-value**	**VIF**	**VIF**
Puffing time, min	<0.0001	0.6226	0.2653	0.2512	1.22	1.32
Average Puff duration, sec	0.5307	<0.01	0.2153	0.2655	1.32	1.32
Average flow rate, ml/sec	0.001272	0.5008	0.2201	0.1762	1.34	1.34
Average inter-puff interval, sec	<0.0001	0.1189	0.1866	0.2396	1.32	1.32
Number of puffs	<0.0001	0.4175	0.2543	0.1441	1.34	1.32
Total inhaled volume, ml	<0.0001	0.2483	0.2434	0.261	1.34	1.32
Average Puff volume, ml	<0.0001	<0.0001	0.1959	0.2517	1.33	1.33
Maximum puff volume, ml	0.003654	0.7236	0.1386	0.1641	1.33	1.33
Nicotine plasma boost	0.000582	<0.0001	0.2893	0.3399	1.20	1.21

© 2024 Ferdous T et al. *JAMA Network Open*.

eReferences

1. Sample Size Software | Power Analysis Software | PASS | NCSS.com. Accessed February 8, 2024. https://www.ncss.com/software/pass/
2. Vargas-Rivera M, Ebrahimi Kalan M, Ward-Peterson M, et al. Effect of flavour manipulation on ENDS (JUUL) users' experiences, puffing behaviour and nicotine exposure among US college students. *Tob Control*. Published online May 23, 2020:tobaccocontrol-2019-055551. doi:10.1136/tobaccocontrol-2019-055551
3. Cohen J. *Statistical Power Analysis for the Behavioral Sciences*. 2nd ed. L. Erlbaum Associates; 1988.

© 2024 Ferdous T et al. *JAMA Network Open*.
