# Supplementary material for: Partial Nicotine Reduction and E-Cigarette Users’ Puffing Behaviors Among Adults Aged 21 to 35 Years: A Randomized Crossover Clinical Trial
Source: JAMA Netw Open. 2024 Jul 26;7(7):e2422954. doi: 10.1001/jamanetworkopen.2024.22954 (PMC11282440; doi:10.1001/jamanetworkopen.2024.22954)
Supplement: Supplement 3. — Data Sharing Statement [file jamanetwopen-e2422954-s003.pdf]

Data Sharing Statement

Ferdous. Partial Nicotine Reduction and E-Cigarette Users' Puffing Behaviors Among Adults Aged 21 to 35 Years. *JAMA Netw Open*. Published July 26, 2024.
doi:10.1001/jamanetworkopen.2024.22954

Data
Data available: Yes
Data types: Deidentified participant data
How to access data: Individual participant data that underlie the results reported in this article can be shared after deidentification (text, tables, figures, and appendices). Any researcher who provides a methodologically sound proposal can have access to the data access. Proposals should be directed to the corresponding author. Data requestors will need to sign a data access agreement to gain access.
When available: beginning date: 09-01-2025

Supporting Documents
Document types: None

Additional Information
Who can access the data: Any researcher who provides a methodologically sound proposal can have access to the data. Proposals should be directed to the corresponding author.
Types of analyses: Any researcher who provides a methodologically sound proposal of analysis can have access to the data.
Mechanisms of data availability: Data requestors will need to sign a data access agreement to gain access.
